# Implementing a multilevel, multicomponent intervention to engage fathers in complementary feeding in Northern Nigeria: Perceptions of deliverers and recipients

**DOI:** 10.1371/journal.pgph.0005214

**Published:** 2025-10-03

**Authors:** Diana Allotey, Valerie L. Flax, Abiodun F. Ipadeola, Sarah Kwasu, Beamlak Worku, Keerti Kalluru, Rami Imam, Angela M. Stover, Sujata Bose, Stephanie L. Martin

**Affiliations:** 1 Department of Nutrition, Gillings School of Global Public Health, University of North Carolina at Chapel Hill, Chapel Hill, North Carolina, United States of America; 2 RTI International, Research Triangle Park, North Carolina, United States of America; 3 Datametrics Associates Limited, Abuja, Nigeria; 4 Alive & Thrive, Kaduna State, Nigeria; 5 Department of Health Policy and Management, Gillings School of Global Public Health, University of North Carolina at Chapel Hill, Chapel Hill, North Carolina, United States of America; 6 Lineberger Comprehensive Cancer Center, University of North Carolina at Chapel Hill, Chapel Hill, North Carolina, United States of America; 7 Alive & Thrive, Washington, District of Columbia, United States of America; 8 Carolina Population Center, University of North Carolina at Chapel Hill, Chapel Hill, North Carolina, United States of America; PLOS: Public Library of Science, UNITED STATES OF AMERICA

## Abstract

The Alive & Thrive multilevel, multicomponent intervention to engage fathers in complementary feeding in Kaduna State, Nigeria previously showed significant increases in complementary feeding practices for children. This analysis explores the perceptions of intervention deliverers and recipients to inform future spread. The intervention components included counseling cards, home visits, feeding bowls, texts/prerecorded messages, posters, leaflets, sermon guides, talking points, radio and television spots. In-depth interviews (24) were conducted with intervention deliverers (community health extension workers, community and religious leaders) and focus group discussions (16) with recipients (parents of children 6–23 months) from 6 rural and urban wards. Participants were purposively sampled; parents were not selected as couples. Eligibility for CHEWs and CRLs included being ≥ 18 years and having participated in intervention implementation. Eligibility for parents included being ≥18 years (or married mothers 15–17 years), having a biological child 6–23 months, and receiving the intervention. Transcripts were coded descriptively in Atlas.ti and the results were mapped to the domains and constructs of the Consolidated Framework for Implementation Research 2.0. For the innovation domain, intervention deliverers and recipients reported high acceptability and appropriateness of the intervention components. For the outer domain, the intervention was perceived to be influenced by values and beliefs (fathers’ roles as providers), systemic conditions (economic hardships), and critical incidents (COVID-19). The intervention was also influenced by relational connections, compatibility, intervention deliverers and recipients, teaming, tailoring strategies and engaging for the domains of inner setting, individuals, and implementation process, respectively. For implementation strategies, intervention deliverers liked the training and monthly meetings where they shared experiences and problem solved. The Alive & Thrive intervention in Kaduna State, Nigeria was acceptable, appropriate, and feasible for intervention deliverers and recipients.

## Introduction

Adequate complementary feeding is critical to meeting the nutritional requirements for health, growth, and development of children 6–23 months of age. The World Health Organization (WHO) established global guidelines for caregivers to feed infants and young children [1]. In Nigeria, like other low- and middle-income countries (LMICs), sub-optimal complementary feeding practices prevail with low national prevalence of key complementary feeding indicators – minimum dietary diversity (23%), minimum meal frequency (42%), and minimum acceptable diet (11%) [2]. In Nigeria, mothers are the predominant caregivers of infants and young children, [3,4] but family members, such as grandmothers and fathers, influence complementary feeding practices. [5–7] Similar to many communities [8–10], in Kaduna State in Northern Nigeria, fathers’ roles consist primarily of providing money and food for complementary feeding. [5,11]

Social and behavior change (SBC) interventions to improve complementary feeding have utilized varied approaches to engage influential family members in complementary feeding, including home visits, peer couple groups, father groups, community meetings, text messaging, mass media, or a combination. [12] Intervention studies have reported improved feeding practices, increased father involvement, and increased support from fathers for childcare and feeding. [5,13–15]

In Kaduna State, the Alive & Thrive initiative implemented a multilevel, multicomponent SBC intervention to include fathers in complementary feeding. [11] After one year of implementation, there were significant increases in children’s consumption of fish and eggs, minimum meal frequency, and minimum acceptable diet; fathers’ and mothers’ complementary feeding knowledge; and fathers’ provision of money for complementary foods, but no significant changes in children’s dietary diversity scores or minimum dietary diversity. [11] Exposure to intervention components was low among mothers and fathers (11% and 26% respectively); mothers’ exposure to SBC activities was positively associated with complementary feeding, but fathers’ exposure was not. [11] However, a higher level of father complementary feeding support was positively associated with minimum meal frequency, minimum acceptable diet, and feeding children fish. [5]

Given that low participation in activities to engage men in infant and young child feeding (IYCF) can be a challenge, [14,16–18] more research is needed to understand the experiences of innovation deliverers and recipients. This analysis sought to understand deliverers’ and recipients’ perspectives on the implementation of the Alive & Thrive intervention, implementation barriers and facilitators, and the acceptability, appropriateness and feasibility of intervention components. Results are organized by Consolidated Framework for Implementation Research (CFIR) 2.0 domains to increase the applicability to other contexts.

## Methods

### Study setting

The intervention was implemented in 6 administrative wards (4 rural: Igabi, Kwarau, Turunku, and Zangon Aya; 2 urban: Rigachikun and Rigasa,) out of the 12 total of Igabi local government area in Kaduna State, Nigeria. The region comprises a mixture of people of diverse ethnicities and religions, with Hausas and Fulanis being the two predominant ethnic groups, while Islam and Christianity are the dominant religions. [19–21] Administrative wards were purposefully selected to represent the blend of urban and rural, and ethnic and religious diversity of Kaduna State. Complementary feeding practices are sub-optimal in the state. Among children aged 6–23 months in Kaduna State, 51.0% met minimum meal frequency, 15.6% met minimum dietary diversity, and 13.9% meet the minimum acceptable diet. [22]

### Intervention implementation

From July 2019 to July 2020, the Alive & Thrive initiative and I Care Women and Youth Initiative (ICARE) designed and implemented an intervention in Igabi local government area to promote optimal complementary feeding practices by increasing fathers’ involvement, and mothers’ knowledge, skills, and social support through interpersonal communication, community mobilization, and mass media. [11] The focus on fathers’ involvement was influenced by formative research that identified fathers as “a high-potential untapped audience for improving infant and young child feeding practices.” [23] Intervention components included counseling cards for community-based organizations (CBOs) and community health extension workers (CHEWs) (**Table 1**). The CBOs used the counseling cards during community meetings and CHEWs used them during home visits to reinforce complementary feeding messages with mothers and fathers (when present). Community and religious leaders (CRLs) used talking points and sermon guides containing complementary feeding messages during community events and religious services. Alive & Thrive developed the sermon guides for use during religious services or religious gatherings such as naming ceremonies and weddings where fathers were present. [24] CHEWs provided mothers with feeding bowls and leaflets that showed nutritious foods and the quantity of food to feed infants and children at different ages. Fathers with mobile phones received weekly text messages and prerecorded voice messages. Complementary feeding messages were displayed on posters and broadcast through radio and TV spots.

**Table 1:** Intervention components and implementation strategies.

Intervention components received by recipients	Implementation strategies*
Counseling cards Home visits Talking points Sermon guides Feeding bowls Leaflets Posters Text messages and prerecorded voice messages Radio and tv spots**	Training for CBOs, CHEWs and CRLs - Make training dynamic Monthly learning collaborative meetings for CHEWs and CRLs - Create a learning collaborative - Implementation team meetings - Promote network weaving Develop and distribute educational materials Revise professional roles

* *Strategies used to train, educate and develop interrelationships among the deliverers.*

**Complementary feeding SBC messages were broadcast to mothers and fathers through radio and TV spots

As part of implementation strategies, ICARE trained 13 CBOs, 18 CRLs and 60 CHEWs on intervention components and messages in 2019, at the start of the intervention. ICARE held separate monthly meetings for CHEWs and CRLs throughout the implementation period to reinforce training content, discuss challenges, and brainstorm solutions (**Table 1**). In March-April 2020, government COVID-19 restrictions suspended home visits and religious/community meetings, while digital messaging and mass media continued. [25]

### Study design, eligibility criteria and sampling methods

The data used in this analysis were collected post-intervention in September 2020 as part of a larger program evaluation (clinicaltrials.gov NCT04835662). CRLs and CHEWs were eligible to participate in in-depth interviews (IDIs) if they were ≥ 18 years and participated in intervention implementation. CRLs and CHEWs were purposively sampled to reflect diversity in their experiences. CRLs were sampled to ensure variation in position, years of experience, and ward; CHEWs were selected to reflect differences in years of experience and ward. Trained research assistants contacted those individuals by phone or in person (if no phone number was available), described the study, and asked if they were willing to participate. Those who consented to participate were interviewed in person.

Mothers and fathers were eligible to participate in focus group discussions (FGDs) if they were ≥18 years, with a biological child 6–23 months, and received the intervention. Mothers were also eligible if they were 15–17 years and married. The sampling process for the FGDs involved dividing each of the six wards into three sections in consultation with community leaders. A purposive sample of four eligible mothers and four eligible fathers who had participated in ICARE SBC activities were recruited from each of the three sections, totaling 12 eligible mothers and 12 eligible fathers per ward. Mothers and fathers were not selected as couples. Out of 12 mothers and 12 fathers recruited, 10 each participated in the separate mother and father FGDs per ward, with the remaining two designated as “back ups” (in case other recruited individuals decided not to participate). Four FGDs (two for mothers, two for fathers) were conducted in each urban ward and two FGDs (one for mothers, one for fathers) were conducted in each rural ward.

### Ethical approval

The Kaduna State Ministry of Health and the RTI International institutional review boards provided ethical approval. Written informed consent was obtained from all participants.

### Data collection

IDIs and FGDs were conducted mostly in Hausa with a few in English by Nigerian male and female research assistants; both were led by a moderator and supported by a notetaker. All research assistants and notetakers lived in Kaduna State and spoke Hausa, a dominant language in the region. They were college-level educated with at least 5 years of research experience and training in qualitative research methods and the study protocol.

Separate interview guides were used for IDIs with CRLs and CHEWs and included questions on their roles in child health and nutrition, and their experiences promoting the intervention, including barriers and facilitators. The guides also included questions about their perceptions about the training, support, and resources they received from Alive & Thrive (S1 Text).

Separate FGD guides were used with mothers and fathers. The FGD guide for mothers included questions on their exposure to and perceptions of intervention components, their motivations and experiences incorporating intervention messages into their feeding practices, and their perceptions and experiences with fathers’ involvement in child feeding and care. The FGD guide for fathers included questions on their exposure to and perceptions of intervention components and their experiences with child feeding and care (S2 Text).

IDIs lasted 30–45 minutes and FGDs lasted 1 to 1.5 hours and recorded using digital voice recorders. All participants received 2000 Naira (approximately $5) as compensation.

### Data analysis and CFIR constructs

IDIs and FGDs were transcribed verbatim, translated into English, and imported into Atlas.ti (version 9.0). A two-stage analytic approach was employed. Stage 1 involved descriptive coding to capture participants’ experiences and perspectives. Two analysts coded the IDIs, and three coded the FGDs. A hybrid deductive-inductive approach guided coding. [26] Deductive codes were developed based on the interview guides and existing literature, while inductive codes emerged directly from the data. Each transcript was coded independently by two analysts and any discrepancies were resolved through consensus. Descriptive coding of the IDIs and FGDs occurred concurrently, with regular meetings between the two teams to compare findings across data sources. For the IDIs, the coded text segments were grouped into descriptive categories and summarized in matrices with illustrative quotes. FGD data were examined with Atlas.ti co-occurrence queries with Boolean operator “AND” to explore combinations of codes [27] and summaries with illustrative quotes were prepared for each descriptive category. In Stage 2, analysts collectively reviewed the descriptive categories, and by consensus, systematically mapped them to the relevant domains and constructs of the Consolidated Framework for Implementation Research (CFIR 2.0), [28] which provided the interpretive structure for reporting results. CFIR is a meta-theoretical framework to identify factors that influence intervention implementation. [28,29] Implementation strategies were defined based on Powell et al. [30] Proctor et al’s definitions of acceptability, feasibility, and appropriateness were used to examine implementation outcomes. [31]

## Results

A total of 24 in-depth interviews (IDIs) with 12 female CHEWs and 12 male CRLs and 16 FGDs, 8 each with mothers and fathers were conducted. The CRLs included 6 community leaders (e.g., village heads, CBO leaders), 5 imams, and 1 pastor. The CHEWs and CRLs had 6 months to 10 years and 15 months to 30 years of experience in their roles, respectively. The findings from the IDIs and FGDs are organized by all CFIR 2.0 domains and 11 of the constructs:

1. Innovation domain: innovation design
2. Outer setting domain: values and beliefs, systemic conditions, and critical incidents
3. Inner setting domain: relational connections and compatibility
4. Individuals domain: innovation deliverers and innovation recipients
5. Implementation process domain: teaming, tailoring strategies, and engaging

**Fig 1:**
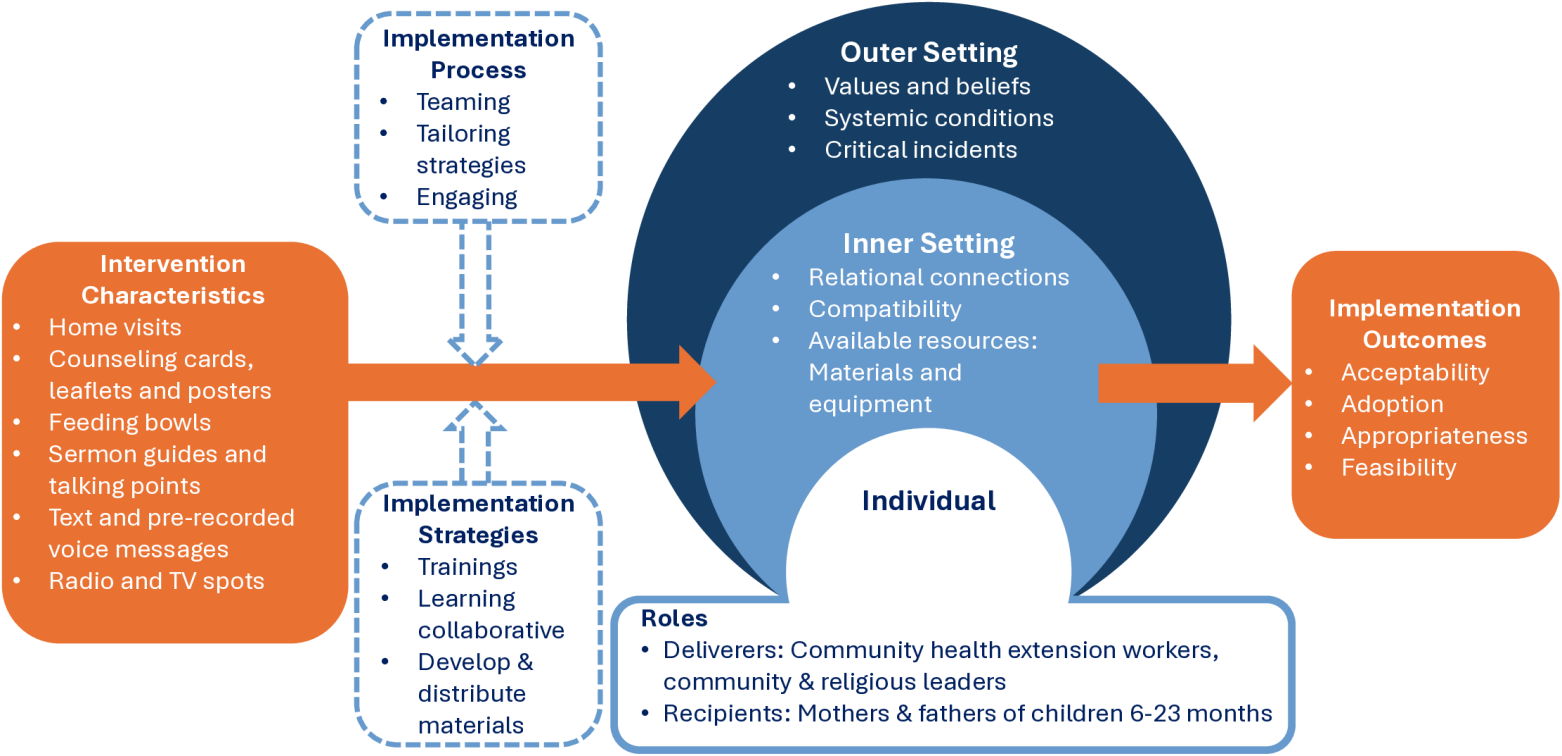
CFIR domains and constructs, and implementation outcomes of the Alive & Thrive study in Kaduna State, Nigeria.

We also include acceptability, appropriateness and feasibility of implementation strategies (training and monthly learning collaborative meetings) and deliverers’ recommendations to improve the innovation. **Fig 1** represents a synthesis of these CFIR domains, constructs and implementation outcomes [32].

### Innovation domain

#### Construct: Innovation design.

*Home visits.* CHEWs reported home visits were helpful for communicating intervention messages to families. Mothers appreciated CHEWs’ visits. On the infrequent occasions that fathers were present during home visits, they reported positive experiences, which CHEWs confirmed. Some mothers said that when CHEWs speak directly to fathers about complementary feeding it was more acceptable to fathers than mothers relaying the messages from the CHEWs. Fathers who interacted with CHEWs during home visits reported that CHEWs talking with fathers during home visits encouraged mothers to take CHEWs’ advice. “*We really want the CHEWs to come around because if they meet the women and she is not serious about the CHEWs’ advice, when mothers see the fathers accept the CHEWs’ advice, they will also accept it.” —*Rigasa, Father.

*Counseling cards, leaflets, and posters.* Most CHEWs and CRLs appreciated the simplicity of the counseling cards, leaflets, and posters given to them for use during home visits, meetings, and religious services, which helped communicate complementary feeding messages. “*The poster we were given really helped us a lot as it makes it easy to communicate*.” —Kwarau, CRL. Mothers and fathers appreciated the leaflets and posters because they were attractive and easy to understand, even for people with low literacy. *“I like it because it is easy to understand. All the foods that are supposed to be fed to a child are on the poster and ways to prepare it are there, too.” —*Kwarau, Mother*.*

*Feeding bowls*. Most CHEWs reported positive experiences distributing feeding bowls to families and encouraging mothers to use them to feed their children. A few CHEWs mentioned the insufficient amounts of bowls and asked that more be made available: “*The help we need now is the bowl. The mothers want the bowls because that is what will help them learn how to feed their children. When they have the bowls, it makes it easy for them and those bowls also help us with our work…We need more bowls*” —Kwarau, CHEW. Many mothers reported positive experiences using the bowls. They appreciated the illustrations of food and graduations in the bowl that helped them know the quantity of food to feed their children. However, some mothers reported the bowls broke easily, and a few fathers reported that not all mothers in polygynous households received feeding bowls. *“They give our wives these bowls. But when they enter some households that have more than one wife that has children between 6 and 9 months and give just one bowl, they face issues because the women get angry and say why do you give her and not give me, too?”* —Turunku, Father.

*Sermon guides and talking points.* CRLs reported using and valuing the sermon guides and talking points with their congregations and communities to promote optimal infant and young child feeding and fathers’ involvement. “*I use the manual during sermons to teach the congregation. I memorize the easy parts so that I can know what to tell people at the mosque. I make sure to carry the manual along to the mosque for the parts that are hard, put it inside the book I use to preach and relate it to them at the right time. —*Turunku, CRL.

*Text messages and prerecorded voice messages*. Most fathers appreciated the complementary feeding text messages and prerecorded voice messages. Fathers were happy with the convenience of being able to easily share text messages because they forwarded them to other people. A few fathers reported preferring text messages over prerecorded voice messages: “*To me, these [text]messages are still a way of passing the information to people in case one didn’t hear it either from the groups or in the mosques from the imam, or the information did not reach one’s community, or his house was not visited. But with the text message, anywhere you are, you will receive it at your own comfort and convenience*.” —Zangon Aya, Father.

*Radio and TV spots.* CHEWs reported the intervention messages broadcasted on radio and TV reinforced the messages’ significance to parents: “*A parent will think he knows it and will not believe what you are telling him but when he hears the same thing being said on the radio, he will say this is what this person has been telling me, and this encourages them to practice [the messages]*.” —Turunku, CHEW.

### Outer setting domain

***Construct: Values and Beliefs***. Social norms about fathers’ roles as providers were widespread and all CHEWs and CRLs agreed that it was important to involve fathers in complementary feeding. *“Fathers should always be involved because they are the ones that provide the food for the household. If only the woman is informed and she tells her husband, he might not listen or accept it.”* —Kwarau, CRL. Participants also described the importance of including CRLs based on the importance of religion in their communities. “*Our community doesn’t accept new things without religion being included. So, I use both religious and health perspectives to teach them because both talk about parents’ responsibilities with child feeding.”* —Rigachikun, CRL

***Construct: Systemic conditions***. CHEWs and CRLs reported that many families faced economic hardships limiting their ability to feed diverse foods to their children. Some CHEWs mentioned understanding parents’ economic burdens and tailoring counseling strategies to promote available indigenous nutritious foods. *“For some parents, when we go to their houses, they will be complaining that they don’t have money. But we CHEWs tell the parents that it is not something that is far from their community.”* —Zangon Aya, CHEW.

***Construct: Critical incidents.*** A few CHEWs and CRLs mentioned that the COVID-19 pandemic had exacerbated families’ existing financial barriers, and some families asked for money in addition to advice about feeding their children diverse foods. *“The barrier is that most people in our community are poor and because of the present situation in the country, Corona [COVID-19] we have so many people that are poorer than before.*” —Rigasa, CRL. Some CHEWs and CRLs also noted that the monthly meetings were paused during the pandemic. “*Before we were called every month for a meeting and if we attend, we were given 2000 naira but after COVID-19 came they stopped calling us.*” —Rigasa, CRL. Mothers also reported that home visits were paused.

### Inner setting domain

***Construct: Relational connections.*** Implementing the intervention using CHEWs and CRLs who were well-known to community members helped make the intervention feasible and ensure successful implementation. CRLs reported that including people who knew them and could attest to their credibility was helpful when counseling people they did not know. “*I have a good relationship with them. They are not strangers to me, and I am not to them. If it happens that I am going to a place where they don’t know me, I do take along people whom I have advised before and they will testify to that*.” —Igabi, CRL. Most CRLs reported that their existing leadership roles in the community and their cordial relationships with community members facilitated their roles as deliverers. “*It is easier because of my position in society. Being an imam and a teacher, they believe I won’t lie to them or deceive them. So, my influence is key to convincing them*.” —Turunku, CRL.

***Construct: Compatibility***. Most religious leaders reported the ease of incorporating complementary feeding messages into their sermons and teachings. *“On Fridays when I climb the altar, I draw people’s attention and encourage them with Quran verses and scriptures about how to feed their children certain foods they need at 6–8 months, 9–11 months, and 11 months upwards.”* —Rigasa, CRL.

### Individuals domain

***Construct: Innovation deliverers – CHEWs and CRLs.*** CHEWs described their roles were to promote IYCF in their communities and conduct home visits with mothers and when present, fathers. Most CHEWs mentioned giving health talks or providing IYCF counseling broadly, but others specifically mentioned tailoring counseling strategies to complementary feeding and encouraging mothers to feed their children diverse foods.

The CRLs explained their role related to child nutrition was to advise parents on child feeding and included specific recommendations about feeding beans, vegetables, and animal source foods. They agreed on the importance of talking with fathers about complementary feeding. “*They are the head of the family so if you get them [fathers], you have gotten the family*.” —Rigasa, CRL. Most CRLs reported fathers were receptive to their messages because they referred to passages from religious texts (Quran/Bible). “*I will use the Quran verse which was given to encourage them on their responsibilities to feed and care for these children.”* —Rigachikun, CRL. Some CRLs reported that a few fathers were critical of their messages while others were curious and asked a lot of questions. “*You know sometimes men are so critical that they ask a lot of questions before they accept the program.*” —Kwarau, CRL.

***Construct: Innovation recipients* – *Parents of children 6–23 months of age****.* Mothers reported that they appreciated that child feeding messages were incorporated in the community gatherings for fathers. “*The advice is good…it makes my husband put in more effort buying food for the family*.” —Rigachikun, Mother. Fathers also said they liked hearing about child feeding during their community meetings. Both mothers and fathers said that receiving advice from religious leaders was helpful because religious leaders were influential with both mothers and fathers. “*Some men don’t usually listen to their wives, but sharing the information with them [fathers]at the mosques makes them [fathers] accept it*.” —Rigasa, Mother.

Many fathers also mentioned learning about the foods needed to ensure their children were fed diverse diets. They spoke about the locally available foods that were easy to obtain and how important it was to incorporate them as part of existing recipes for meals they commonly consumed. *“Even if it’s our traditional ones that aren’t expensive, like soya beans. When it is prepared, it builds the body well. Things from irrigated farming, like carrots, spinach, and tomatoes, are no longer expensive now.*” Igabi, Father.

### Implementation process domain

***Construct: Teaming.*** Several CHEWs reported coordinating and working closely with the CRLs. CHEWs said that CRLs often paved the way by ensuring the CHEWs were welcomed, which enabled them to speak freely with mothers and fathers, making the intervention feasible. “*When we enter the community…we meet the community leader because the leader knows his community very well. He will talk to them and tell them so and so person is in the community to work for you and for your children. Please give your cooperation to them, so when we enter the houses, we don’t have problems with the mothers. They will give attention and even the fathers also give attention to us.*” —Zangon Aya, CHEW.

***Construct: Tailoring strategies****.* While most CHEWs reported positive experiences during home visits, a few reported challenges with families’ expectations of financial support to purchase recommended foods. Most CHEWs reported addressing mothers’ financial concerns by encouraging them to use locally available foods and tailoring their recommendations to fit the context*.* A few CHEWs reported a shortage of bowls and families that did not receive the bowls were reluctant to meet and listen to them during home visits. Some CHEWs reported encouraging those families to use bowls similar to the feeding bowls to feed their children. *“We also encourage those that don’t have the bowl to use another clean bowl to feed their children.”*—Kwarau, CHEW.

***Construct: Engaging***. Although very few CHEWs talked about meeting with fathers during the home visits, among those who did, most described positive experiences engaging with fathers about feeding their children diverse diets. They reported that some fathers now know which nutritious foods to buy for their children. CHEWs also reported that after talking to them, fathers became motivated to provide recommended foods. “*When you reach a community, you will find fathers sitting outside. Even at the community that I went to, I didn’t have to go to the mothers, I just stopped at the gathering of fathers. And what makes me happy when I deliver the message is that the fathers promise me that by God’s grace, next visit, that I’m going to see the thing that I mentioned to them. The next visit I went, I saw those things they were doing.*” —Zangon Aya, CHEW. Fathers said hearing the messages from multiple sources motivated them to try the messages. “*At first when we were called, I thought we were the only ones they called. After a little while, I now heard the imam making an announcement and giving an explanation about it. I also heard it on the media and got a text on my phone. So that motivated me.*” — Kwarau, Father.

*Commitment of the implementers. S*ome mothers and fathers reported that CHEW’s persistence motivated them to apply the recommendations. “*When I saw them coming every time, I never took them seriously. I sat down one day and thought that it must be very important for them to come always.*” —Rigachikun, Father.

*Communication skills*. Most CHEWs reported using a friendly and open communication style to engage parents during home visits, which encouraged mothers to be receptive to their recommendations.

### Implementation strategies

***Training*.** Almost all the CHEWs and CRLs appreciated the training they received because it gave them skills to incorporate complementary feeding messages into lessons on parenting during home visits, meetings, and religious services. “*I find it easy because I was adequately trained on what to tell them. I confidently do it because I know what to say to them due to my training.”* —Kwarau, CRL. Many CHEWs said the role plays during training were very useful and they valued the sessions that taught them how to use the print materials (leaflets) and the feeding bowls when counseling mothers and fathers.

***Monthly meetings***. CHEWs and CRLs valued the monthly meetings, which served as a learning collaborative. Most CHEWs reported that they appreciated the reinforcement of key messages they had received during training. “*It’s very helpful because some of the things we learnt, we forget them, but we are reminded during the meetings*.” —Zangon Aya, CHEW. Other CHEWs also said the monthly meetings provided them with opportunities to share experiences, strategies, and problem solve. “*The helpful side is that during the meetings we were able to tell them the problems we were facing in the households we were visiting. We told them our views and we were all able to discuss how we were going to achieve our goals*.” —Rigasa, CHEW. Some CHEWs mentioned that they appreciated refreshments provided during the meetings and the transportation reimbursement.

### Innovation deliverers’ recommendations for innovation improvements

***Include other family members*.** CHEWs and CRLs recommended involving grandmothers and older children, as well as grandfathers, aunts, uncles, and co-wives. Almost all the CHEWs and CRLs reported that involving more family members would be beneficial and emphasized that in most homes, there are other family members apart from mothers who care for and feed children. “*All should be involved in complementary feeding - father, mother, grandmother, grandfather and caregivers…They should be involved because the father brings the food for the mother or the caregiver or the grandmother, who prepares the food*.”—Rigachikun, CHEW. Some CHEWs reported engaging with other family members, especially grandmothers, specifically during home visits to ensure that the whole family was aware of the complementary feeding recommendations. They mentioned that in certain circumstances having an additional family member hear the message in addition to the mother strengthened the credibility of the message when mothers communicated the recommendations to fathers. “*The grandmothers also help. I may meet the mother. When the father comes back and the wife explains to him, he may ask her to leave him alone…But if the grandmother knows about it, when he is complaining, she will tell him it’s not a lie…So, you see the grandmother has a strong stake in being involved*.”—Turunku, CHEW.

***More opportunities to learn from one another*.** Some CHEWs and CRLs communicated their desire for more frequent meetings with the project team to provide opportunities for them to share their challenges and problem-solve in a timely manner. There were also some CHEWs who recommended that they should be given more opportunities to collaborate with CRLs to be able to engage more fathers. The community leaders also requested that more religious leaders be included in future complementary feeding interventions. “*The improvement should be that the number of pastors and imams should be increased.”* Rigasa, CRL*.*

***Increase intervention materials and logistical support*.** Most CHEWs and CRLs recommended an increase in the quantity of intervention materials, especially the feeding bowls, posters, and leaflets, because due to the limited quantities, not all families had received them. A few CRLs suggested improving the quality of the educational materials to ensure they lasted longer and were not easily destroyed when exposed to weather conditions. “*The sun can beat them and also rain, so with time they will get spoilt. So, we should have more of them.*” Igabi, CRL. Some CHEWs and CRLs also suggested an increase in the allowance they were given for their work. “*Based on our last meeting with other imams and pastors, we suggested that more posters should be printed, and the meeting should be done more frequently, and monthly stipends should be increased.”* —Igabi, CRL.

## Discussion

Using relevant CFIR 2.0 domains and constructs, we found that Alive & Thrive’s multilevel, multicomponent intervention to engage fathers in complementary feeding was feasible, appropriate and acceptable for both innovation deliverers (CHEWs and CRLs) and innovation recipients (parents). Intervention deliverers and recipients identified opportunities to strengthen intervention components and implementation. Deliverers found that the implementation strategies (training and monthly meetings) were feasible, appropriate and acceptable and provided opportunities to develop relationships, share experiences, and problem solve.

### Innovation design

Innovation deliverers reported positive experiences implementing the intervention components and appreciated the ease of use and simplicity of the intervention materials making the intervention feasible. Similarly, most innovation recipients appreciated the complementary feeding messages and support they received from the CHEWs and CRLs and the intervention materials, text messages, and prerecorded voice messages. Recipients found complementary feeding messages to be easy to understand and, for most, easy to implement. However, innovation deliverers and recipients reported challenges around fathers’ participation in home visits, financial constraints for some families to practice complementary feeding recommendations, and frustration with limited feeding bowl supplies. Fathers’ limited participation in home visits in this study reinforce findings from the impact evaluation, which found that fathers rarely interacted with CHEWs during home visits [11]. Intervention studies to engage fathers in nutrition in other LMICs also have noted fathers’ low participation [16–18,33,34]. In Kaduna, fathers’ support of complementary feeding is limited to their traditional roles [7], but fathers increasing their support beyond their traditional roles was associated with improved complementary feeding practices [5]. CHEW visits are traditionally scheduled with pregnant women and mothers of young children. Expanding these visits to effectively include fathers likely requires intentional scheduling [35,36] and tailored counseling that reflect fathers’ roles [37].

Participants suggested including grandmothers, older children, and other family members in the intervention in addition to fathers. This recommendation is consistent with calls to use a family systems approach to improve maternal, infant, and young child nutrition [38], and the influence of social norms on grandmothers’ and fathers’ roles in complementary feeding. [39] There are multiple examples of interventions to engage grandmothers in nutrition [12], yet examples that engage older siblings are lacking [35], despite their roles in infant and young child care and feeding [40].

### Outer setting

Local economic conditions limited some families’ ability to partake in the recommended complementary feeding actions. Cost is a commonly reported barrier to complementary feeding recommendations [41], however some CHEWs responded to these concerns by tailoring their recommendations to promote indigenous and accessible foods to families. The COVID-19 pandemic also resulted in temporary pauses to monthly meetings by CHEWs and CRLs and services provided at the community-level such as home visits. Having multiple communication channels that did not require in-person contact such as radio and TV to transmit the intervention messages was useful in mitigating the restrictions of COVID-19.

Social norms about fathers’ roles as providers and the expectation that they should purchase or provide complementary foods [5] facilitated intervention implementation. In the impact evaluation of this intervention, most mothers reported that fathers were the people who most influenced complementary feeding, and, at endline, more fathers provided money, purchased complementary foods, gave advice about child feeding, and participated in child feeding [11]. Increasingly, interventions to engage fathers in maternal and child health and nutrition use gender transformative approaches that promote equity and seek to change gender relations. [33,42,43] This intervention used a gender-neutral approach, which promoted fathers’ involvement by considering underlying gender norms and relations rather than attempting to address them. Future interventions could consider using gender transformative approaches to increase women’s agency, improve nutrition, and strengthen family relationships. [33,44]

### Inner setting

Innovation deliverers valued having regular, facilitated meetings to share challenges and problem solve together, similar to father and grandmother peer educators in Kenya. [45] Program guidance for engaging fathers in nutrition highlights the importance of providing opportunities for innovation deliverers to collectively share experiences and problem solve. [33]

For CRLs, delivering the intervention was feasible because it was compatible with their leadership roles, and they could integrate complementary feeding and parenting recommendations into their sermons and community activities. CRLs have been trained to influence complementary feeding norms elsewhere. [39] For CHEWs, this compatibility with roles was limited because home visits typically focus on mothers and could not easily engage fathers as part of their ongoing activities.

### Implementation process

Deliverers’ intentional coordination of activities and CRLs’ introductions of and support for CHEWs in the community facilitated implementation. CHEWs reported tailoring recommendations to families’ concerns and using a friendly communication style strengthened their interactions during home visits. Tailoring can enhance recipients understanding and acceptance of recommended practices. [37,46]

### Limitations

The CFIR was useful for the analysis and interpretation of our data, but we did not use the CFIR to design the data collection tools [47], which may have led to the omission of relevant topics. Hence, some of the CFIR constructs (e.g., innovation source) were not reflected in our findings. Also, only mothers and fathers who participated in the intervention were included in FGDs, and thus it is possible that parents who chose not to participate would have important perspectives on intervention acceptability and feasibility. However, our focus was on implementation, and it was important to examine the recipients’ perspectives. We recommend future IYCF SBC studies use the CFIR 2.0 framework throughout the process of intervention design and evaluation.

Despite these limitations, using CFIR 2.0 constructs proved to be useful in identifying barriers and facilitators to implementation and understanding the intervention components that were acceptable, feasible, and appropriate to both innovation deliverers and recipients. Since the conceptualization of the original CFIR, implementation research studies in both high-income countries (HIC) and LMICs have used this “determinants framework” to identify factors that influence program implementation and outcomes. [48,49] Systematic reviews on the use of the CFIR in HIC [48] and LMIC [49] settings revealed that the majority of studies that have used this framework have been in healthcare settings with less frequent use in community-based settings. [48] A literature search identified very few studies that have used CFIR constructs to assess the implementation of community-based IYCF interventions in LMICs. [50,51]

The qualitative methods used for this research allowed in-depth exploration of the innovation’s implementation through triangulation of responses across multiple participant categories. While participants shared both challenges and frustrations, suggesting a level of comfort in expressing positive and negative experiences, some responses may have been influenced by social desirability bias, potentially leading to an overstatement of the innovation’s success. This potential bias should be considered when interpreting the results.

## Conclusions

The Alive & Thrive intervention to engage fathers in complementary feeding in Kaduna State, Nigeria was acceptable, appropriate and feasible for intervention deliverers and recipients. Deliverers and recipients recommended scheduling home visits around men’s schedules, providing frequent opportunities for CHEWs and CRLs to meet, ensuring adequate supplies of counseling materials, and engaging other family members to improve program impact. Future research should include fathers during the intervention design phase to identify strategies to increase fathers’ sustained participation, monitor for any unintended consequences related to engaging fathers in complementary feeding, use a family systems approach that includes all influential family members in intervention components and research, and include the perspectives of parents who did not participate in the intervention. The Alive & Thrive intervention provides an example of engaging fathers through multiple communication channels.

## Supporting information

S1 Text In-depth interview guide. (DOCX)

S2 Text Focus group discussion guides. (DOCX)

S3 Text Focus group discussion summaries. (DOCX)

S1 Table In-depth interview summary matrix. (XLSX)

S1 Checklist Inclusivity in global research. (DOCX)
