# Supplementary material for: Implementing a multilevel, multicomponent intervention to engage fathers in complementary feeding in Northern Nigeria: Perceptions of deliverers and recipients
Source: PLOS Glob Public Health. 2025 Oct 3;5(10):e0005214. doi: 10.1371/journal.pgph.0005214 (PMC12494235; doi:10.1371/journal.pgph.0005214)
Supplement: S1 Text — (DOCX) [file pgph.0005214.s001.docx]

**S1. File IN-DEPTH INTERVIEW GUIDE**

**RELIGIOUS AND COMMUNITY LEADERS IN INTERVENTION AREAS**

Thank you for talking with me today. We are interested in learning from religious and community leaders who are part of the infant and young child feeding program in this community. We will ask you questions about your experience and opinions related to supporting families in your area. I’m happy to be learning from you. We are talking with you to learn about religious and community leaders’ experiences promoting optimal young child feeding practices. There are no right or wrong answers— you’re the expert. Your answers will be the best way for us to understand the current situation.

**Begin Recording**

Can you tell me about your position?

- How long have you been in it?
- What are your responsibilities?

What is your role related to child health and nutrition?

- How often do you talk with members of your congregation/community about how to feed children 6-23 months of age?

How did you come to participate in the infant and young child feeding program in this LGA?

What advice do you give to families with children 6-23 months of age about how to feed their children?

What are your experiences with holding sermons or meetings in the community to talk with fathers about improving the diet of their young children?

**(Probe)**: What made it easy for you to talk with fathers about improving the dietary diversity {or how young children are fed} of their young children?

The infant and young child feeding program has encouraged fathers to improve the diet diversity of their young children. What is a diverse diet for children age 6-23 months?

What were the barriers to talking with fathers about improving the dietary diversity of their young children?

What additional support would you like to help you talk with fathers about dietary diversity of their young children?

What do you think about encouraging fathers to be more involved in improving the dietary diversity of their children?

- How have fathers reacted to this advice?
- How have mothers reacted?
- What are positive aspects of engaging fathers?
- What are negative aspects of engaging fathers?
- Are there situations where you do not think it makes sense to involve fathers? [If so] what are those situations?
- Are there other family members that should also be involved in improving the dietary diversity of young children?
- Who?
- Why do you think they should also be involved?

What, if any, changes have you noticed among families in your congregation/community related to improving dietary diversity of their young children?

- What makes it easy for fathers to improve child dietary diversity?
- What makes it difficult for fathers to improve child dietary diversity?

What, if any, changes have you made in the feeding of your own children after you got involved in this program?

What should be the role of religious leaders to improve feeding of young children?

**Training**

When you think back to the training you received on feeding young children 6-23 months of age, how satisfied are you with the quality of the training you received? Why?

- What aspects of the training did you find most useful?
- Is there anything that you wish had been included? [If so] what else should have been included?
- Anything else you would like to learn? [If so] what else would you like to learn?
- Are there any topics or issues that you still have questions about? [If so] what are they?

What skills and information that you received from the training do you use in your role supporting families to practices recommended complementary feeding practices?

What situations have you encountered that the training did not prepare you for?

What materials do you have to help you counsel families of young children 6-23 months about complementary feeding?

- How do you use these materials?
- What do you like about them?
- What don’t you like about them?

**Support and Resources**

Can you describe the support you received from the infant and young child feeding program in your efforts to encourage fathers to be more involved in improving child dietary diversity?

- How has this support been helpful?
- How could it be more helpful?

Do you participate in the monthly review meetings?

- Can you describe these meetings? What happens during these meetings?
- What works well?
- What is helpful?
- What could be improved?
- Is there any type of support you would like to receive that you are not currently getting?

Based on your experience talking about complementary feeding as part of your sermons and community activities, do you think religious leaders and CBO leaders/staff in other areas could encourage fathers to support recommended complementary feeding practices? Why or why not?

Thank you for taking the time to talk with us and share your experiences. Do you have any final thoughts or experiences to share about how young children are fed?

 Page Break

**IN-DEPTH INTERVIEW GUIDE: COMMUNITY HEALTH EXTENSION WORKERS IN INTERVENTION AREAS**

Thank you for talking with me today. We are interested in learning from CHEWs who are part of the infant and young child feeding program in this community. We will ask you questions about your experience and opinions about advising families in your area. I’m happy to be learning from you. We are talking with you to learn about CHEW’s experiences. There are no right or wrong answers— you’re the expert. Your answers will be the best way for us to understand the current situation.

**Background**

How long have you been a CHEW?

How were you selected/identified to be a CHEW?

Could you tell me about your role as a CHEW? (**Probe)**: How often do you conduct home visits? Which types of families or what geographic area do you visit?

When you visit a home, who in the family do you usually meet with?

What do you think are the main challenges that families face related to feeding young children 6-23 months of age in your community?

**Complementary feeding**

What advice do you give to families with children 6-23 months of age about how to feed their children?

What are your experiences with making home visits to talk with families about improving the diet of their young children?

- What made it easy for you to talk with families about improving the dietary diversity of their young children?
- What were the barriers to talking with families about improving the dietary diversity of their young children?
- What additional support would you like to help you talk with families about dietary diversity of their young children?

What do you think about encouraging fathers to be more involved in improving the dietary diversity of their children?

- How have mothers reacted to this advice?
- How have fathers reacted?
- What are positive aspects of engaging fathers?
- What are negative aspects of engaging fathers?
- Are there situations where you do not think it makes sense to involve fathers? [If so] what are those situations?

Are there other family members that should be involved in complementary feeding?

- Who?
- Why should they be involved?

In the last year, what, if any, changes have you noticed among families you visit related to the dietary diversity of their children 6-23 months of age?

- What makes it easy for families to practice these recommendations?
- What makes it difficult for families to practice complementary feeding recommendations?

What do you think about the recommendation encouraging families to add eggs or mashed soft fish to mashed sweet potatoes or mashed pumpkins and add mashed beans or mashed spinach?

- Do you suggest this to the families that you work with? Why or why not?
- How do families react to this suggestion?
- What feedback have families who have tried this recommendation given you?

**Training**

From which organization(s) have you received training on feeding children 6-23 months of age in the last year?

- When you think back to that training, how satisfied are you with the quality of the training you received? Why?
- What aspects of the training did you find most useful?
- Is there anything that you wish had been included? [If so] what else should have been included?
- Anything else you would like to learn? [If so] what else would you like to learn?
- Are there any topics or issues that you still have questions about? [If so] what are they?

What skills and information that you received from the training about feeding young children 6-23 months do you use in your role as a CHEW?

What situations have you encountered that the training about feeding young children 6-23 months did not prepare you for?

What materials do you have to help you counsel families of young children 6-23 months about complementary feeding?

- How do you use these materials?
- What do you like about them?
- What don’t you like about them?

**Support and Resources**

Can you describe the supervision you receive from the PHC In Charge?

- How has this supervision been helpful?
- How could it be different so that it would be more helpful?
- Is there any type of support you would like to receive that you are not currently getting?

Do you participate in meetings on infant and young child feeding?

- How often do you participate in these meetings?
- Can you describe these meetings? What happens during these meetings?
- What works well?
- What is helpful?
- What could be improved?

Based on your experience counseling families on complementary feeding, do you think CHEWs in other areas could encourage fathers to engage in child dietary diversity? Why?

Do you think engaging fathers in child dietary diversity is an appropriate recommendation for families in this area? Why/why not?

How do we ensure that CHEWs continue to sustain this IYCF program?

Thank you for taking the time to talk with us and share your experiences. Do you have any final thoughts or experiences to share about how young children are fed?
