# Supplementary material for: Implementing a multilevel, multicomponent intervention to engage fathers in complementary feeding in Northern Nigeria: Perceptions of deliverers and recipients
Source: PLOS Glob Public Health. 2025 Oct 3;5(10):e0005214. doi: 10.1371/journal.pgph.0005214 (PMC12494235; doi:10.1371/journal.pgph.0005214)
Supplement: S2 Text — (DOCX) [file pgph.0005214.s002.docx]

**S2. File** **FOCUS GROUP DISCUSSION GUIDES**

**FATHERS OF CHILDREN 6-23 MONTHS INVOLVED WITH THE INTERVENTION**

**INSTRUCTIONS:**

1. Introduce yourself and the purpose of the focus group
2. Confirm that each participant has consented to participate.  Summarize what consenting to participate means.
3. Explain how a focus group works and agree upon ground rules

- Only one person should speak at a time so we can hear everyone’s opinion. There may be a temptation to jump in when someone is talking, but please wait until they have finished.
- There are no right or wrong answers. We are not trying to test anyone’s knowledge or ability.
- You do not have to speak in a particular order.
- You don’t have to agree with the views of others in the group. You can express your own view. But please do so respectfully. We are interested in hearing different honest views.
- Please keep this discussion private and do not repeat what you hear to people outside of this group.

1. Ask if they have any questions
2. Ask each participant to introduce themselves by telling their name and the age of their child
3. Remind them that you will be recording the discussion and taking notes

**Begin Recording**

| ROLES AND RESPONSIBILITIES | |
| --- | --- |
| A1 | What should a young child be fed to have adequate, nutritious food?  **(Probe):**What kinds of foods and liquids do young children 6-23 months need to have adequate, nutritious food?  Why? |
| A2 | Who in a family is responsible for seeing that a young child is fed adequate, nutritious food? **(Probe)**:  Anyone else?  What do they do? (Ask about grandmothers if not mentioned) |
| A3 | What is a father’s role in making sure his young child is fed adequate, nutritious food? |
| A4 | What difficulties do mothers face when it comes to caring for and feeding a young child age 6-23 months?  **(Probe):**What help and support do mothers need to overcome these difficulties?  Who can provide this help? |
| VIGNETTE | |
| B1 | Thank you for your participation in the discussion so far. I will now tell you a story of a family and would like you to help fill in the story with your ideas.    Within this region there is a small village where a man named Yusuf was living happily with his wife, Aisha, and their first child Laraba, a girl. Laraba was one year old. Aisha, the mother, was taking care of cooking, household chores, running her business as a small trader, and caring for Laraba. Her workload often kept her busy while she was at home or kept her away from home during the day. She found it very difficult to find time to do all of her tasks. She also worried about having enough good foods for Laraba to grow well and stay healthy. Often Laraba only ate plain pap for her meals.”      **(Stop here. Ask the following questions):**   - If Aisha, the mother, is taking care of most of the household chores and a small business, what do you think Yusuf, the father, is doing each day? **(Probe):** What else? In what ways does the father support feeding Laraba? - What do you think are the good foods that Laraba should be receiving to grow well and stay healthy? - What will happen to Laraba if she continues to be fed only plain pap? **(Probe):** Why? - What can be done so that Laraba is better fed?   **(If participants make suggestions that we would not recommend, such as giving biscuits or cheese balls, ask why they think that would be good, etc. without agreeing to the suggestion)** |
| B2 | **(Continue with the story)**  …"One day Yusuf ‘s brother Kabiru who lived in a nearby town visited Yusuf‘s family. Everybody in the community liked Kabiru. Aisha and Yusuf were very happy to receive Kabiru. Kabiru was happy to see the child. In fact, it was Kabiru who suggested the name for Laraba when she was born. Kabiru noticed that Laraba seemed thinner and less active compared to when he visited the previous month. He observed how Aisha was overloaded with several chores and unable to spend much time feeding and caring for the child. Kabiru thought of his own very healthy baby who was now 14 months old. Kabiru learned how to feed his child during an event held by a community-based organization he is involved with. Kabiru talked to his brother about the importance of giving Laraba the right kinds of foods to eat.  He explained how important this was and that everyone in a family can help feed and care for their children.  Kabiru came up with some suggestions for Yusuf to help Laraba eat better…”    **(Stop here and ask the questions below on possible solutions):**  What suggestions do you think Kabiru will give to his brother?   - Which ones do you think Yusuf will agree to? **(Probe):** Why? - Which suggestions will be difficult for Yusuf to try? Why?  1. How could that problem be solved?  - What else could Yusuf do to help be sure Laraba is eating well? **(Probe):** Anything else? - What could others in the family do to help ensure baby Laraba is eating well? **(Probe):** Anything else? |
| B3 | Based on our discussion today, please tell me in your own words, how will the story end?  **(Ask several different men to suggest their own endings to the story.)** |
| B4 | Thank you for your good ideas about this story. I’d now like you to think about your own families and your own experiences. What actions do you, as a father, take related to the feeding and care of your child? |
| INFANT AND YOUNG CHILD FEEDING PROGRAM ACTIVITIES | |
| C1 | During the last 6 months, there have been several activities in your community recommending ways for fathers to become more involved in how children ages 6-23 months are fed. Are you aware of any of these activities happening in your community?  **If yes:**     - Which ones? - Have you participated in any of these activities? Which ones? - Did you convince other people to attend? If so, who? |
| C2 | How many of you are members of a community-based organization?    **If any are members:**  During the meetings of your organization(s) in the last 6 months, did anyone talk about ways for fathers to become more involved in how children ages 6-23 months are fed?  **For participants who DID hear someone from a CBO talk about child feeding:**   - Who from the community-based organization talked about feeding young children? **(Probe)**on the role of the person in the organization? - What did they recommend? What is your opinion of those recommendations? **(Probe)**on what they liked and didn’t like. - What new information did you learn about child feeding during the meetings? - What is your opinion about having someone talk with fathers about child feeding during your CBO meetings? **(Probe)**on what they liked and didn’t like. - What are your experiences with trying to apply the recommendations in your families? **(Probe)**on what worked and didn’t work and why they did or didn’t try the recommendations. - What motivated you to apply the recommendations when feeding your child?   **For participants who DID NOT hear anyone from a CBO talk about child feeding in the last year**:   - What is your opinion about having someone talk with fathers about child feeding during a community-based organization meeting? |
| C3 | In the last 6 months, have you heard religious leaders talking about how to feed children 6-23 months of age?  **For participants who DID hear a religious leader talk about child feeding:**   - What did they recommend? - What is your opinion of those recommendations? **(Probe)**on what they liked and didn’t like. - What new information did you learn about child feeding from religious leaders - What is your opinion about having religious leaders talk about child feeding during their sermons? **(Probe)**on what they liked and didn’t like. - What are your experiences with trying to apply the recommendations in your families? **(Probe)**on what worked and didn’t work and why they did or didn’t try the recommendations. - What motivated you to apply the recommendations when feeding your child?   **For participants who DID NOT hear a religious leader talking about child feeding in the last year**:   - What is your opinion about having religious leaders talk about child feeding during their sermons? |
| C4 | In the last 6 months, has your family been visited by a CHEW?    **For participants who WERE visited by a CHEW:**   - What did they recommend related to feeding your young child?      - What is your opinion of those recommendations? **(Probe)**on what they liked and didn’t like. - What is your opinion about having CHEWS talk about child feeding during their visits to your home? **(Probe)**on what they liked and didn’t like. - What are your experiences with trying to apply the recommendations in your families? **(Probe)**on what worked and didn’t work and why they did or didn’t try the recommendations. - What motivated you to apply the recommendations when feeding your child? - Were you at home when the CHEW visited? - **If yes,** what new information about child feeding did you learn from the CHEW? - **If not,** would you like the CHEW to schedule visits when you are home? Why/why not?   **For participants whose families WERE NOT visited by a CHEW in the last year**:   - What is your opinion about having CHEWs talk about child feeding during home visits? |
| C5 | In the last 6 months, have you received calls or messages on your mobile phone about how to feed young children age 6-23 months?  **For participants who HAVE received calls or messages on their mobile phone:**   - What did the calls or messages say? - What is your opinion of the messages themselves? **(Probe/prompt with part of message)**on what they liked and didn’t like. - What new information about child feeding did you learn from the messages? - What is your opinion about receiving messages on young child feeding through your phone? **(Probe)**on what they liked and didn’t like. - What are your experiences with trying to apply the recommendations from the messages in your families? **(Probe)**on what worked and didn’t work and why they did or didn’t try the recommendations. - What motivated you to apply the recommendations when feeding your child?   **For participants who HAVE NOT received calls or messages on their mobile in the last year**:   - What are your opinions about receiving messages on young child feeding through your phone? |
| C6 | **[Show the Dietary Diversity poster.]** Have you seen this poster in the last 6 months?    **If yes:**   - Where have you seen it? - What is it asking you to do? - What do you like about this poster? - What do you not like about this poster? - Do you think this poster is meant for people like you? Why or why not?   **For participants seeing the poster for the first time**:   - What is this poster asking you to do? - What do you like about this poster? - What do you not like about this poster? - Do you think this poster is meant for people like you? Why or why not? |
| C7 | **[Show the Dietary Diversity reminder pamphlet.]** Have you seen this pamphlet in the last 6 months?    **If yes:**   - Where have you seen it? - What is it asking you to do? - What do you like about this pamphlet? - What do you not like about this pamphlet? - Do you think this pamphlet is meant for people like you? Why or why not?   **For participants seeing the pamphlet for the first time**:   - What is this pamphlet asking you to do? - What do you like about this pamphlet? - What do you not like about this pamphlet? - Do you think this pamphlet is meant for people like you? - Why or why not? |
| REACTIONS TO FATHER ENGAGEMENT | |
| D1 | The infant and young child feeding program in your area suggests that fathers can oversee how their children are fed.   - What do you think about this advice? - What makes it easy for fathers to be involved and oversee how children are fed? - What makes it difficult for fathers to oversee how children are fed? - What does your wife think about this advice? |
| D2 | The infant and young child feeding program in your area suggests that fathers can procure nutritious foods like eggs, fish, pumpkins, sweet potatoes, spinach, and beans to be fed to their 6-23-month-old child.   - What do you think about this advice? - What makes it easy for fathers to purchase foods for the young child? - What makes it difficult for fathers to purchase foods for the young child? - What does your wife think about this advice? |
| D3 | The infant and young child feeding program in your area suggests that fathers can make sure mothers have enough time to feed their 6-23-month-old child.   - What do you think about this advice? - What makes it easy for fathers to give time for their wives to feed the young child? - What makes it difficult for fathers to give time for their wives to feeding the young child? - What does your wife think about this advice? |
| D4 | The infant and young child feeding program in your area suggests that fathers can procure soap for handwashing.   - What do you think about this advice? - What makes it easy for fathers to purchase soap? - What makes it difficult for fathers to purchase soap? - What does your wife think about this advice? |
| CLOSING | |
|  | Thank you for taking the time to talk with us and share your experiences. Does anyone have any final thoughts or experiences to share about fathers becoming more involved in how young children are fed? |

Page Break

**FOCUS GROUP DISCUSSION GUIDE:**

**MOTHERS OF CHILDREN 6-23 MONTHS WHOSE FATHER WAS INVOLVED WITH THE INTERVENTION**

**INSTRUCTIONS:**

1. Introduce yourself and the purpose of the focus group
2. Confirm that each participant has consented to participate.  Summarize what consenting to participate means.
3. Explain how a focus group works and agree upon ground rules

- Only one person should speak at a time so we can hear everyone’s opinion. There may be a temptation to jump in when someone is talking, but please wait until they have finished.
- There are no right or wrong answers. We are not trying to test anyone’s knowledge or ability.
- You do not have to speak in a particular order.
- You don’t have to agree with the views of others in the group. You can express your own view. But please do so respectfully. We are interested in hearing different honest views.

Please keep this discussion private and do not repeat what you hear to people outside of this group.

1. Ask if they have any questions
2. Ask each participant to introduce themselves by telling their name and the age of their child
3. Remind them that you will be recording the discussion and taking notes

**Begin Recording**

| ROLES AND RESPONSIBILITIES | |
| --- | --- |
| A1 | What should a young child be fed to have adequate, nutritious food? **(Probe):**What kinds of foods and liquids do young children 6-23 months need to have adequate, nutritious food? Why? |
| A2 | Who in a family is responsible for seeing that a young child is fed adequate nutritious food? **(Probe)**: Anyone else? What do they do? (Ask about grandmothers if not mentioned)   - What is a father’s role in making sure his child is fed adequate nutritious food? - What is a mother’s role in making sure her child is fed adequate nutritious food?   **(Probe)**on how feeding is related to having a child who grows well and is healthy. |
| COMPLEMENTARY FEEDING PRACTICES | |
| B1 | - What is easy about feeding a young child 6-23 months of age? - What is difficult about feeding a young child 6-23 months of age? |
| B2 | What difficulties do mothers face when it comes to caring for and feeding a young child 6-23 months of age?   - What help and support do mothers need to overcome these difficulties? - Who can provide this help? |
| B3 | In your opinion, what types of foods should be given to a child 6-23 months to make sure they have a variety of foods?   - What makes it difficult to feed children a variety of foods? - What makes it easier? |
| **SOURCES OF ADVICE ON CHILD HEALTH AND FEEDING** | |
| C1 | What advice on child feeding have you received in the last 6 months?   - Where have you received information or advice on child feeding? - What do you think about this advice? - What are your experiences with applying the advice you received about child feeding?   **(Follow up questions should be applied to each piece of advice shared by participants)** |
| C2 | In the last 6 months, has a CHEW come to your house to talk with you about feeding your young child?  **For participants who WERE visited by a CHEW:**   - How was their visit? How often did they come? - What did the CHEW recommend related to feeding your young child? - What is your opinion of those recommendations? **(Probe)** on what they liked and didn’t like. - What new information did you learn about child feeding from the CHEW? - What is your opinion about having CHEWs talk about child feeding during their visits to your home? **(Probe)** on what they liked and didn’t like. - What are your experiences with trying to apply the recommendations in your families? **(Probe)** on what worked and didn’t work and why they did or didn’t try the recommendations. - What motivated you to apply the recommendations when feeding your child? - Was your husband there during the CHEWs visit?   **If Yes:**   - What were your experiences with having your husband participant in the meeting with the CHEW? - What were your experiences if your husband was present for the meeting? - How did he react when you shared information from the CHEW?   **For participants who WERE NOT visited by a CHEW in the last year**:   - What is your opinion about having CHEWs talk about child feeding during home visits? |
| C3 | In the last 6 months, have you heard religious leaders talking about how to feed young children 6-23 months of age?    **For participants who DID hear a religious leader talk about child feeding:**   - What do they recommend? - What is your opinion about having religious leaders talk about child feeding during their sermons? **(Probe)** on what they liked and didn’t like. - What new information about child feeding did you learn from the sermons? - What are your experiences with trying to apply the recommendations in your families? **(Probe)** on what worked and didn’t work and why they did or didn’t try the recommendations. - What motivated you to apply the recommendations when feeding your child?   **For participants who DID NOT hear a religious leader talking about child feeding in the last year**:   - What is your opinion about having religious leaders talk about child feeding during their sermons? |
| C4 | In the last 6 months, have you heard or seen any information on the radio, television, or social media about how to feed your young child 6-23 months of age?  **If yes:**   - What information have you heard or seen? - Which parts of the information, if any, were new? - Where did you hear or see this information (radio, television, social media)? - What do you like about this information? - What do you not like about this information? - Have you made any changes to how you feed your young child as a result of this information? |
| C5 | **[Show the Dietary Diversity poster.]** Have you seen this poster?  **If yes:**   - Where have you seen it? - What is it asking you to do? - What do you like about this poster? - What do you not like about this poster? Why? - How, if at all, has this influenced how you feed your 6-23 month old?   **For participants seeing the poster for the first time**:   - What is this poster asking you to do?                What do you like about this poster?   - What do you not like about this poster?                 Do you think this poster is meant for people like you? Why or why not? |
| C6 | **[Show the Dietary Diversity reminder pamphlet.]** Have you seen this pamphlet?  **If yes:**   - Where have you seen it? - What is it asking you to do? - What do you like about this pamphlet? - What do you not like about this pamphlet? Why? - How, if that all, has this influenced how you feed your 6-23 month old?   **For participants seeing the pamphlet for the first time**:   - What is this pamphlet asking you to do? - What do you like about this pamphlet? - What do you not like about this pamphlet? - Do you think this pamphlet is meant for people like you? Why or why not? |
| C7 | **[Hold up the complementary feeding bowl.]** Have you seen this bowl?  **If yes:**   - Where have you seen it? - Do you have this bowl? - Who gave it to you? - How do you use this bowl? - What do you like about this bowl? - What do you not like about this bowl? Why? |
| C8 | Have you heard of the advice to add eggs or mashed soft fish to mashed sweet potatoes or mashed pumpkins and add mashed beans or mashed spinach for children 6-23 months of age?   - What do you think about this advice? - Have you tried this advice? Why or why not? - What would make it easy to feed a young child this food? - What would make it difficult to feed a young child this food? |
| FATHER INVOLVEMENT | |
| D1 | How is your husband involved in feeding your 6-23 month old child? What is his role?   - What does your husband do to help with feeding your 6-23 month old? - What do you like about how your husband is involved? - What would you like to be different about how your husband is involved? |
| D2 | There have been activities in your community and to individual men to involve fathers in feeding young children 6-23 months of age. Are you aware of these activities?   - What can you tell me about these activities? **(Probe)** on CBO meetings on fathers’ involvement in child feeding, religious sermons, text and voice messages - How has your husband participated in these activities? - Has his participation influenced how your 6-23 month old child is fed? - Have you noticed any differences in his involvement in young child feeding since he participated in these activities? If so, how? - Have you noticed any other kinds of differences at home since your husband participated in these activities? What types of differences? |
| CLOSING | |
|  | Thank you for taking the time to talk with us and share your experiences. Does anyone have any final thoughts or experiences to share about how young children are fed? |

Page Break
