# Supplementary material for: Implementing a multilevel, multicomponent intervention to engage fathers in complementary feeding in Northern Nigeria: Perceptions of deliverers and recipients
Source: PLOS Glob Public Health. 2025 Oct 3;5(10):e0005214. doi: 10.1371/journal.pgph.0005214 (PMC12494235; doi:10.1371/journal.pgph.0005214)
Supplement: S3 Text — (DOCX) [file pgph.0005214.s003.docx]

**Summaries for FGDs with mothers and fathers**

**Ward name: Igabi**

*Note: For each of the questions, note if it was similar or different between the groups of fathers and mothers from each ward. Include any quotes that are particularly relevant*

**PERCEPTIONS**

Ques 1: How did fathers/mothers in the area describe their thoughts/opinions/likes and dislikes (**perceptions)** of the recommendations made by the program (CBOs, CHEWs, Religious leaders, phone calls/text messages, pamphlets, posters, feeding bowl, rec. dietary pattern) about feeding their children?  **Hint: look out for use of Perceptions + Exposure codes**

**What were the similarities?**

**CBOs: N/A. Only fathers spoke about their thoughts**

**CHEWs**: Both mothers and fathers spoke positively about the advice they received from the CHEWs.

“Moderator: What is opinion about having CHEW talking about child feeding during their visit to your home?

Respondents: It is very good and important thing. Glory be to God, it is a good thing for them to visit us so that they can further enlighten us about things we do not know so that we can use it and improve our lives

Respondent: like he just said, everything is true. Even if you are not around and someone comes to talk to your family about good food, you will practice it.

Respondent: glory be to god. It is a good thing to hear about nutritious foods from a health personnel. It is not a topic you play with.”----Fathers FGD

**Feeding bowl: N/A. only mothers mentioned this**

**Pamphlet**: N/A. Only mothers mentioned this

**Phone calls/text messages**: N/A. Only fathers mentioned this

**Posters:** Both mothers and fathers had positive thoughts about the posters. Mothers spoke about the general healthful appearance of the illustrated children on the posters as being attractive.

**“**Moderator: How did the poster draw your attention to feed up your child between 6months- 23 month?

Respondent 5: The picture draw my attention. Because of the foods on the poster.

Respondent 6: The children are in good health. The things given to the child makes the child look healthy and we would like to do same.

Respondent 2: All people draw my attention. Everything on the poster and the foods listed are very important. That is why we were asked to give to our children.

Respondent 4: All people draw my attention. Everything on the poster and the child eating fascinates me because the child is healthy.

Respondent 7: All people draw my attention. Everything on the poster. That is why we try it and reap its benefits.

Respondent 9: All people draw my attention. Because we admire everything on the poster. That is why we use it.

Respondent 1: All people draw my attention. Everything on it motivates us to give them.

Respondent 8: All people draw my attention. It’s true. Everything on the poster is what we use.

Respondent: it is like that

Respondent: yes everything on the poster encourages us to give our children.

Moderator: What did you like dislike about the poster?

Respondent 5: Am okay with all.

Respondent 8: Am okay with all, if there is addition they should.

Respondent 7: Am okay with all, if there is addition they should. Everything is okay

Respondent 9: Am okay with all, if there is addition they should. Everything is okay

Respondent 4: Am okay with all, if there is addition they should. Everything is okay

Moderator: How did the poster draw your attention to the feeding of your baby between 6 month to 23 month?

Respondent 2: The baby is looking strong and healthy because of the food he was feed and everything in it. That is why we are giving our children too to eat**.”---Mothers FGD**

**Radio/TV/social media:** None mentioned for mothers and fathers in this community.

**Rec. Dietary pattern:** N/A. Only mothers mentioned this.

**Religious Leaders:** Both mothers and fathers mentioned positive thoughts about hearing religious leaders speak about IYCF.

**“**Moderator: What is your opinion of those recommendations?

Respondent 10: They are all god advice and it will help us in keeping our children, wives and households. They are good and we accept it with both hands and we are trying to practice it in our homes.

Respondent: Honestly, these things that are brought, house heads that know what they are doing know that they are trying to help them, their health, the health of their wives and children. Because when you hear something and tell someone else, there will be improvement. With the advice given, if we will try and like people in the rural area that make mistakes will correct it. Sometimes when they bring children to the hospital, you will barely recognize then as human beings. But with this knowledge gained, the child will not go through such.

Moderator: What new information did you learn from the religious leaders?

Respondent 9: How to take care of our households, wives and our children in the right way. Honestly, many at times our religious leaders really try in the aspect of enlightening us on how to care for our family. From our children to our wives. Especially one imam, after every Morning Prayer, sometimes he stands and enlighten us in the mosque because in Islam there are different type of celebrations. Eid-el Fitr, Eid-el Adhan. Yesterday he reminded us in the Mosque that every Friday he will remind us to always support our families. If you eat rice or corn, get meat and add to your soup. If it is jollof rice, get fish and put in it. Give them eggs and other things that will build their body. They do call our attention to that” -------Fathers FGD.

**What were the differences?**

**CBOs:** Fathers expressed general positive thoughts about receiving advice during their meetings on IYCF and on the new things they learnt

“Moderator: What is your opinion about having someone talk with fathers about child feeding during a community-based organization meeting?

Respondent 2: It is good because it exposes us to things that we suppose to know about child feeding. It is good because every day in life you will get to find out about something you have but you do not know their importance until someone comes to enlighten you about it. It might be a tree in your house that you just use as shade but someone will come and tell you its importance and you will see that it is true. So, the different organizations that come to enlighten the public on these types of things are of great importance and the general public is supposed to accept them with both hands so as to gain more knowledge, improve their lives and prevent diseases from getting to small children in other to save money and improve.”----Fathers FGD

**CHEWs**: N/A. Both had positive thoughts

**Feeding bowl:** Mother spoke general positive thoughts about the feeding bowls. However, most mothers said that the bowls were easy to break which was something they disliked

**“**Moderator: How do you use this plate?

Respondent 2: A child of 6 month should be given food up to red line and at 11 month we should give food at minimum green line while above 23 month we give our baby food that will fill the plate to eat.

Respondent 1: A child of 6 month should be given food up to the redline while 11 month food at green and a baby at 21 month we should fill the plate.

Moderator: What did you like about the plate?

Respondent 8: The plate is good but baby can easily break it while eating.

Respondent 2: The plate can break easily because my only last for 1 month. I wish the plate is metal in nature so that whatever the child does with it, it doesn’t break.

Moderator: What did you dislike about the plate?

Respondent 2: The Plate break easily

Respondent 10: The Plate break easily

Respondent 1: The plate break easily compare to the previous once given to us that is unbreakable.

Respondent: I have something to say. The plate is very good. No matter what a child does with it, it doesn’t break compared to other places. It is very good”.----Mothers FGD

**Pamphlet**: Mothers mentioned liking the illustrations of the pamphlets

“Moderator: What do you like about the flyers?

Respondent 2: We are happy seen these flyers becomes we have improved a lot

Moderator: Why do you like these flyers?

Respondent 2: We make use of the illustrations on the flyers since they brought it.

Respondent 1: We make use of the illustrations and even give people around us and we want the people that haven’t gotten it to get it.

Moderator: What did you dislike about these posters/flyers?

Respondent 2: Nothing

Respondent 1: Nothing”---Mothers FGD

**Phone calls/text messages**: Fathers mentioned general positive thoughts about the phone calls/text messages. Some fathers mentioned though that the text messages were devoid of sender’s details and so they who were in the rural areas rarely read them. They further said that the text messages should not be from a strange number but rather should be identified with the appropriate source’s name.

“Moderator: What is your opinion about receiving messages on young child feeding through your phone?

Respondents: We like the text messages but they should also indicate who the sender is whether it is from alive and Thriving or Icare. My opinion about it is that, we from rural areas barely read messages because we will think it is from network providers. The messages sent by Alive and Thrive do not have heading, a random number will just send you a message. They should be put Heading. Although they put heading in some but some you will just see a random number and you do not know the sender. Organizations are supposed to be putting headings. So it will better if ICARE or Alive and Thrive shows as the heading. “---Fathers FGD

**Posters:** N/A. Both had positive thoughts. See above

**Radio/TV/social media:** None mentioned for mothers and fathers in this community.

**Rec. Dietary pattern:** Mothers spoke positively about the recommended foods.

“Moderator: What are you thought about the advice?

Respondent 8: I only pick the advice; I felt is good for me.

Respondent 1: All advice given is good for us.”---Mothers FGD.

**Religious leaders:** N/A. Both mothers and fathers mentioned positive thoughts. See above.

**EXPERIENCES**

Ques 2: What were fathers/mothers’ thoughts on the things which worked/didn’t work **(experiences**) and the reasons raised for trying/not trying the recommendations made by the program (CBOs, CHEWs, Religious leaders, phone calls/text messages, pamphlets, posters, feeding bowl, rec. dietary pattern)? **Hint: look out for use of Experiences + Exposure codes**

**What were the similarities?**

**CBOs:** N/A. Only fathers spoke of this.

**CHEWs:** N/A. Only mothers spoke of this.

**Feeding bowl:** None mentioned for both fathers and mothers in this area.

**Pamphlets**: None mentioned for both fathers and mothers in this area

**Phone calls/text messages:** None mentioned for both fathers and mothers in this area.

**Posters:** None mentioned for both fathers and mothers in this area

**Radio/TV/social media:** None mentioned for mothers and fathers in this community.

**Rec. Dietary pattern:** N/A. Only mothers mentioned this.

**Religious Leaders:** Both mothers and fathers mentioned positive experiences with applying the recommendations from the religious leaders.

“Moderator: what are your experiences with trying to apply the recommendations in your families?

Respondents: We have applied the recommendations and we have seen great changes in our families. We also see that our children are growing strong and healthy. What I will like to be more thankful on is when they give us these advice, we practice them and we become happy and our families do not have any problems. We are providing for them and they are happy. If you help your family, ways will open for you and blessings will follow you. But if you do not provide, like the clerics say, if you do not provide what your family will eat, you are cheating yourself. If you do not give your children good food and you feed them food that will spoil them stomach, the money you are supposed to use for other things, you will use it for treatment.

Respondent: In general, the type of explanation given to us by health practitioners and religious leaders, we try to practice them in our houses and we have seen a lot of improvement. Our children’s lives have changed both in the body and health and we have rested in aspect of spending money in hospitals on ailments.”---Fathers FGD

**What were the differences?**

**CBOs:** Fathers said that they had had positive experiences with applying the advice from the CBOs particularly in situations when they were short of money. They remembered substitutions for foods like meat which they learnt from the meetings and they were able to provide some other equally healthy foods for their children.

**‘**’ Moderator: What are your experiences with trying to apply the recommendations in families?

Respondent 9: Though I don’t have the money to buy all the nutritious foods but I do my best to provide for my family. Glory be to God. Before, I didn’t used to pay attention to some things because I didn’t have the means. Because I won’t have and say I do not have. The little I get is what I use. There are types of food I was supposed to be providing but because I didn’t have the means to provide those things or even know the things. Before I used to think, until you give meat. But I now got to understand that, if you get soya beans, it works as meat does. If you get spinach too and other things that supplements other too. I now got to understand that in the aspect of child feeding.

**CHEWs:** Mothers mentioned seeing positive results in their current children compared to their older children when they tried the advice received from the CHEWs

“Moderator: What will you say when trying the advice on your family/

Respondent 9: What we have to say is thank you very much, thank you very much and God will bless you and we want to improve. If there are things we are supposed to improve on, we need it.

Respondent: We have nothing to say than to be thankful. May God increase them in ranks? May God bless us, them and our children that they advise us to care for.

Moderator: What went right or wrong on the advice and why?

Respondent 5: We try advice to see if it will work on our children and to note the differences between the new children and the old ones too

Respondent 8: We take to their advice because it went beyond expectation.

Respondent 7: We take to the advice because they said it and to see if it is wrong and it is right. We adhered to it because what they gave advises on are true and thank you may Allah bless you all.

Respondent 4: The adult is good and are say God bless you. Honestly, we have tried what they said and it is true. May God reward you with blessings.

Respondent 6: The advice is good and we are happy. The advice they came to give us, we have tried it and may God reward them with blessings.”---Mothers FGD

**Feeding bowl**: None mentioned for both fathers and mothers in this area.

**Pamphlets**: None mentioned for both fathers and mothers in this area

**Phone calls/text messages:** None mentioned for both fathers and mothers in this area.

**Posters:** None mentioned for both fathers and mothers in this area

**Radio/TV/social media:** None mentioned for mothers and fathers in this community.

**Rec. Dietary pattern:** Mothers mention general positive experiences with the foods recommended.

**“**Moderator: Have you ever tried this advices.

Respondent: yes, I tried it

Respondent: yes, I tried it.

Moderator: why did you try it?

Respondent 2: You have to try advice to see the best you can use. You must try the advice. When some gives advice, you have to try it to see the outcome. We tried the advice given to us and have seen improvements a lot. Thank you

Respondent 8: We tried the advice and it worked thanks. We used it and have seen its importance**.”---**Mothers FGD

**Religious Leaders:** N/A. Both had positive experiences.

**MOTIVATIONS**

Ques 3: How did fathers/mothers describe what motivated them to apply the recommendations from the program (CBOs, CHEWs, Religious leaders, phone calls/text messages, pamphlets, posters, feeding bowl, rec. dietary pattern)? **Hint: look out for use of Motivations + Exposure codes**

**What were the similarities?**

**CBOs:** N/A. Only fathers mentioned this**.**

**CHEWs:** N/A. Only mothers mentioned this

**Feeding bowl:** None mentioned for both fathers and mothers in this area

**Pamphlets**: None mentioned for both fathers and mothers in this area

**Phone calls/text messages:** None mentioned for both fathers and mothers in this area

**Posters:** None mentioned for both fathers and mothers in this area

**Radio/TV/social media:** None mentioned for mothers and fathers in this community

**Rec. Dietary pattern:** None mentioned for mothers and fathers in this community

**Religious Leaders:** N/A. Only mothers mentioned this.

**What were the differences?**

**CBOs:** Fathers said that the fact that the messages were heath related were their motivations to apply them.

**“**Moderator: what encouraged you to feed your children these foods?

Respondent: what encouraged me is that, whatever talk come up about health, because health is life. If there is no health, you will not find life easy. So, the suggestions that were given were health related and I want to be healthy before anything comes. So, I said I should use them because you do not joke with your health.”---Fathers FGD

**CHEWs:** Mothers mentioned that the perceived benefits they heard about the foods encouraged them to try them.

“Moderator: What gave you the courage to try these advices on child feeding?

Respondent 1: Is because we feel the advice is normal and the food are body building food for our baby. What encouraged us more about what they said is because, all the things mentioned are things we farm. We plant them and they grow from the ground. Even you adult can eat it to build your system and avert ailments talk less of a small child. That is why we use this advice.

Respondent 9: It is true because they are all body building food for our baby

Respondent 6: Because the advices are all good and the food are body building food.

Respondent 3: All they told us is correct and we trust them all.”----Mothers FGD

**Feeding bowl**: None mentioned for both fathers and mothers in this area.

**Pamphlets**: None mentioned for both fathers and mothers in this area

**Phone calls/text messages:** None mentioned for both fathers and mothers in this area.

**Posters:** None mentioned for both fathers and mothers in this area

**Radio/TV/social media:** None mentioned for mothers and fathers in this community.

**Rec. Dietary pattern:** None mentioned for mothers and fathers in this community.

**Religious Leaders:** Some mothers said that the general healthful benefits of the foods recommended motivated them to try them.

**“**Moderator: What gives you courage to try these advice on your family?

Respondent 1: Because the food are natural and good for human being. If you do not try it then you have a problem. If you try it, you will see its importance and will be very happy.

Respondent: The food they listed. The food they listed, if they were man made, some people will not eat it. But since they are gotten from trees, egg from chicken, meat from animals, we know where all these are coming from. That was why we understood it and felt encouraged to try it.”---Mothers FGD

**Any other thoughts you would like to share?**

**Ward name: Kwarau**

*Note: For each of the questions, note if it was similar or different between the groups of fathers and mothers from each ward. Include any quotes that are particularly relevant*

**PERCEPTIONS**

Ques 1: How did fathers/mothers in the area describe their thoughts/opinions/likes and dislikes (**perceptions)** of the recommendations made by the program (CBOs, CHEWs, Religious leaders, phone calls/text messages, pamphlets, posters, feeding bowl, rec. dietary pattern) about feeding their children?  **Hint: look out for use of Perceptions + Exposure codes**

**What were the similarities?**

**CBOs:** N/A

**CHEWs**: Both mothers and fathers spoke positively in their perceptions regarding the community health extension workers. They both appreciated the work the CHEWs did for them. There were no negative comments.

"MODERATOR: What is your opinion about those recommendations?

RESPONDENT 4: They are very good recommendations

RESPONDENT 1: I am very comfortable with their recommendations since they are for my good

MODERATOR: What is your opinion about having CHEWs talk about child feeding during their visits to your home?

RESPONDENT 5: It is a very good thing that they are coming to do. Honestly, it makes them very happy. It makes me really happy because we have increased in knowledge. Because even if you have a degree, you cannot know it all

RESPONDENT: if I hear that a health personnel came to my house to enlighten people, not just my wife it also makes me really happy because I feel, they remembered me and came to my house. They tell how take care of my household and it makes me happy that government k now about me, health personnel know about me and I understand better.

RESPONDENT 8: We have nothing to say but a big thank you to the CHEWs who came to talk to us about important things that concern us and our children"

**Feeding bowl:** N/A

**Pamphlet**: N/A

**Phone calls/text messages**: N/A

**Posters:** Both mothers and fathers spoke positively in their perceptions regarding posters. While mothers were vague in explaining why exactly they enjoyed the posters, fathers were more specific, and one father even suggested that the posters be made in leather form, since paper is a delicate material.

"MODERATOR: What do you like about this poster?

RESPONDENT 9: I like it because it is easy to understand. All the foods that are supposed to be fed to a child is on the poster and ways to prepare it too are there. We were given, so we pasted on walls. Like my tea shop, there is one pasted there

RESPONDENT 9: I like it because it helps me to see practically what I have been hearing

MODERATOR: What do you not like about poster?

RESPONDENT 10: Everything about the poster is good except that it is in paper form. Since it is in paper form. Since it is in paper form if it is posted rain will fall on it and spoil it instead it should be made in leather form. We like everything about the posted. Just that it is in paper form and we are in the raining season. If rain touches it, it was tear. It should be made in leather form, that way it will not get squeezed or spoilt. "

**Radio/TV/social media:** N/A

**Rec. Dietary pattern:** N/A

**Religious Leaders:** Both mothers and fathers spoke of their perceptions regarding religious leaders educating on the topic of child feeding, and they all spoke positively on the topic. Fathers provided specifically what they enjoyed about the sermons while mothers were more vague, just stating that they enjoyed everything.

"MODERATOR: What did they recommend? What is your opinion of those recommendations?

RESPONDENT 1: They spoke so much about what a child needs to eat to be healthy and strong and also the place of the father in child feeding and even those who have heard about it before hand and never believed accepted and believed what the facilitators (trainers) from ICARE Foundation said about child feeding. They gave good recommendation and people understood it. Aside the explanation we do for people, they also added more information on child feeding from 6-23 months and people embraced it very well. They also hear announcement in Mosque which they also got to understand very well. "

**What were the differences?**

**CBOs:** Only fathers spoke of their opinions on community-based organizations. All the respondents spoke positively regarding these organizations.

"MODERATOR: What is your opinion about those recommendations?

RESPONDENT 4: They are very good recommendations

RESPONDENT 1: I am very comfortable with their recommendations since they are for my good

MODERATOR: What is your opinion about having CHEWs talk about child feeding during their visits to your home?

RESPONDENT 5: It is a very good thing that they are coming to do. Honestly, it makes them very happy. It makes me really happy because we have increased in knowledge. Because even if you have a degree, you cannot know it all

RESPONDENT: if I hear that a health personnel came to my house to enlighten people, not just my wife it also makes me really happy because I feel, they remembered me and came to my house. They tell how take care of my household and it makes me happy that government k now about me, health personnel know about me and I understand better.

RESPONDENT 8: We have nothing to say but a big thank you to the CHEWs who came to talk to us about important things that concern us and our children"

**CHEWs**: N/A

**Feeding bowl:** Only mothers spoke of their perceptions regarding the feeding bowl. They only provided positive opinions. They like it especially because the children like it.

"Moderator: What do you like about this bowl?

Respondent 5: I like the bowl because the children are attracted to the bowl, that’s what makes them eat the food.

Moderator: What do you not like about this bowl?

Respondent 1: Nothing

Respondent 4: Nothing, everything is in order"

**Pamphlet**: Only mothers spoke of their perceptions regarding the pamphlets. They explained what they liked about the pamphlets and why. They especially appreciated the tips it provided regarding breastfeeding.

**Phone calls/text messages**: Only fathers spoke on their perceptions regarding phone calls and text messages that provided information on child feeding. The fathers provided only positive perceptions. One father recommended that pictures that are found on the posters be sent through text messages as well.

"MODERATOR: What is your opinion of the messages themselves?

RESPONDENT 9: The messages are good and important. There is one, they explained to us that we should feed our children, beans, spinach, fish, egg and even herbs. We should feed children. That is the new thing I feel I learnt from these messages that weren’t mentioned during the training.

RESPONDENT: The messages are good but like people with big phones they can be adding pictures of food like it is on the poster. They should be adding the pictures too.

RESPONDENT 1: The messages are very good because they usually remind me of my duty to see that my child is fed well"

**Posters:** N/A

**Radio/TV/social media:** N/A

**Rec. Dietary pattern:** Only mothers spoke of their opinions on the recommended dietary pattern provided. It has made them happy.

"Moderator: What do you think about this advice?

Respondent 7: When we give them these foods we are always happy

Respondent 8: It is a good advice when we apply it, we will be happy

Respondent 1: When we started, we were not informed about it, when they tell you, you will think it is rubbish they are saying. But now that we were advised, we will try it"

**Religious leaders:** N/A

**EXPERIENCES**

Ques 2: What were fathers/mothers’ thoughts on the things which worked/didn’t work **(experiences**) and the reasons raised for trying/not trying the recommendations made by the program (CBOs, CHEWs, Religious leaders, phone calls/text messages, pamphlets, posters, feeding bowl, rec. dietary pattern)? **Hint: look out for use of Experiences + Exposure codes**

**What were the similarities?**

**CBOs:** N/A

**CHEWs:** Both mothers and fathers shared their experiences with CHEWs. Mother’s shared their gratitude for the program and explained their experiences with their husbands being present in the meetings and their involvement with child feeding. Fathers explained how they learned about child feeding and hygiene.

"Moderator: What were your experiences with having your husband participate in the meeting with the CHEW?

Respondent 4: Meeting and discussing with him was useful, what they explained to him was useful, he now cares for the child and what the child will eat that will benefit the child.

Moderator: What were your experiences if you husband was present for the meeting?

Respondent 8: He should support with the ways to care for the children so that the child will be healthy.

Respondent 10: When they came to our house, he was there. They discussed with him on how to feed the young children, they explained and he was listening to them and with the recommendations, we have seen changes and the father buys these things for the children because he also heard it from the Religious leaders.

Respondent 9: He understood them and he has continued to do what they asked him to do for the child. We have achieved a lot

Moderator: Thank you"

**Feeding bowl:** N/A

**Pamphlets**: N/A

**Phone calls/text messages:** N/A

**Posters:** N/A

**Radio/TV/social media:** N/A

**Rec. Dietary pattern:** N/A

**Religious Leaders:** N/A

**What were the differences?**

**CBOs:** Only fathers shared their experiences with community-based organizations.

"MODERATOR: What are your experiences with trying to apply the recommendations in your families?

RESPONDENT 4: At first, I had no means of getting the money to buy those nutritious foods for my child but I later started buying and see great improvements in my child"

**CHEWs:** N/A

**Feeding bowl**: N/A

**Pamphlets**: N/A

**Phone calls/text messages:** Only fathers shared their experiences with receiving phone calls and text messages. One respondent shared what he learned from the messages he was receiving.

"MODERATOR: What new information about child feeding did you learn from the messages?

RESPONDENT 8: I also learned that aside using egg, beans, fish and vegetables to feed my child, I must also use fruit"

**Posters:** N/A

**Radio/TV/social media:** N/A

**Rec. Dietary pattern:** N/A

**Religious Leaders:** Only the mothers shared their experiences with religious leaders. They shared what they learned and how they were advised.

"Moderator: What new information about child feeding did you learn from the sermons?

Respondent 4: We have information that is useful because they always tell us to care for our young children and to prepare what the children will eat in a healthy way and always cover their food when they are not eating.

Moderator: Thank you

Moderator: What are your experiences with trying to apply the recommendations in your family?

Respondent 9: Just the way they advised us, we should be giving the children food, it will develop their brain and will also make them intelligent because good food makes them intelligent, we have tried and seen it

Respondent 10: The religious leaders also know about foods that builds the body and also nutritious food is good because anything that will develop the brain and body is what we need

Moderator: Thank you"

**MOTIVATIONS**

Ques 3: How did fathers/mothers describe what motivated them to apply the recommendations from the program (CBOs, CHEWs, Religious leaders, phone calls/text messages, pamphlets, posters, feeding bowl, rec. dietary pattern)? **Hint: look out for use of Motivations + Exposure codes**

**What were the similarities?**

**CBOs:** Only fathers mentioned this

**CHEWs:** Both mothers and fathers shared what motivated them into applying the recommendations given to them by the community health extension workers. Mothers were motivated by their desire to see their children strong and in good health while fathers were motivated by the knowledge the CHEWs had, as well as their determination to help.

"MODERATOR: What motivated you to apply these recommendations when feeding your child?

RESPONDENT 9: I was motivated because I heard the Imam talk about it, I heard people talk about it and I heard it on the radio so, I knew if is very important.

RESPONDENT: what motivated me about the training is, when it started when ICARE came to look for us. Like me that they assigned to be going street to street to take down parent’s names of children of 6-23 months. I knew the importance but it didn’t motivate me but when the people I enlightened started calling me, they call me in some area and I go to educate them and that has really motivated me a lot.

RESPONDENT: What motivated me was, at first when we were called and they explained to us how to feed small children. I thought we were the only ones they called. After a little while, I now heard Imam making an announcement and giving explanation about it. I was surprised and said it is not a playing matter. After he explained, I also heard it on the media and after then I also got a text on my phone. So that motivated me and people we enlighten because they also get text messages. They agreed that this thing is important. It motivated them and me too 100%.

RESPONDENT: what motivated us on this thing is, all these things they asked us to eat are things we normally eat. We just do not know how to prepare them properly and its importance. If it had changed our type of food, it would have been a problem. We used to waste these foods, but we were called to order and thought the right way. That’s the truth of the matter. If it had changed the type of food we eat, it would have caused a lot of troubles. "

**Feeding bowl:** None mentioned by mothers and fathers in this area.

**Pamphlets**: Only mothers mentioned this

**Phone calls/text messages:** None mentioned by mothers and fathers in this area

**Posters:** None mentioned by mothers and fathers in this area

**Radio/TV/social media:** None mentioned by mothers and fathers in this area

**Rec. Dietary pattern: Only mothers mentioned this**

**Religious Leaders:** Both mothers and fathers shared what motivated them into apply the recommendations of the religious leaders. Fathers were motivated by previous organizations and the religious leaders themselves while mothers were motivated by the mere fact that they knew it would help.

"MODERATOR: What motivated you to apply these recommendations when feeding your child?

RESPONDENT 9: I was motivated because I heard the Imam talk about it, I heard people talk about it and I heard it on the radio so, I knew if is very important.

RESPONDENT: what motivated me about the training is, when it started when ICARE came to look for us. Like me that they assigned to be going street to street to take down parent’s names of children of 6-23 months. I knew the importance but it didn’t motivate me but when the people I enlightened started calling me, they call me in some area and I go to educate them and that has really motivated me a lot.

RESPONDENT: What motivated me was, at first when we were called and they explained to us how to feed small children. I thought we were the only ones they called. After a little while, I now heard Imam making an announcement and giving explanation about it. I was surprised and said it is not a playing matter. After he explained, I also heard it on the media and after then I also got a text on my phone. So that motivated me and people we enlighten because they also get text messages. They agreed that this thing is important. It motivated them and me too 100%.

RESPONDENT: what motivated us on this thing is, all these things they asked us to eat are things we normally eat. We just do not know how to prepare them properly and its importance. If it had changed our type of food, it would have been a problem. We used to waste these foods, but we were called to order and thought the right way. That’s the truth of the matter. If it had changed the type of food we eat, it would have caused a lot of troubles."

**What were the differences?**

**CBOs:** Only fathers shared what motivated them into applying the recommendations of the community-based organizations. They were motivated by their past and by their desire to see a better future for their children.

"MODERATOR: What motivated you to apply the recommendations when feeding child?

RESPONDENT 6: I lost one of my children because of malnutrition. So, when I learnt about child’s feeding, I was determined to apply it on my children.

RESPONDENT 7: I was motivated to do more because I saw tremendous changes in my child’s health and growth after I started feeding him with things like milk water, fruits and vegetables"

**CHEWs:** both mentioned positive things which motivated them. See above.

**Feeding bowl**: None mentioned by mothers and fathers in this area.

**Pamphlets**: Only mothers shared what motivated them into applying the information provided on the pamphlets. They were motivated by the information provided by the pamphlet.

"MODERATOR: What motivated you to apply these recommendations when feeding your child?

RESPONDENT 9: I was motivated because I heard the Imam talk about it, I heard people talk about it and I heard it on the radio so, I knew if is very important.

RESPONDENT: what motivated me about the training is, when it started when ICARE came to look for us. Like me that they assigned to be going street to street to take down parent’s names of children of 6-23 months. I knew the importance but it didn’t motivate me but when the people I enlightened started calling me, they call me in some area and I go to educate them and that has really motivated me a lot.

RESPONDENT: What motivated me was, at first when we were called and they explained to us how to feed small children. I thought we were the only ones they called. After a little while, I now heard Imam making an announcement and giving explanation about it. I was surprised and said it is not a playing matter. After he explained, I also heard it on the media and after then I also got a text on my phone. So that motivated me and people we enlighten because they also get text messages. They agreed that this thing is important. It motivated them and me too 100%.

RESPONDENT: what motivated us on this thing is, all these things they asked us to eat are things we normally eat. We just do not know how to prepare them properly and its importance. If it had changed our type of food, it would have been a problem. We used to waste these foods, but we were called to order and thought the right way. That’s the truth of the matter. If it had changed the type of food we eat, it would have caused a lot of troubles."

**Phone calls/text messages:** None mentioned by mothers and fathers in this area

**Posters:** None mentioned by mothers and fathers in this area

**Radio/TV/social media:** None mentioned by mothers and fathers in this area

**Rec. Dietary pattern:** Only mothers shared what motivated them into applying the recommended dietary pattern. They were motivated by the progress of their children.

"MODERATOR: What motivated you to apply these recommendations when feeding your child?

RESPONDENT 9: I was motivated because I heard the Imam talk about it, I heard people talk about it and I heard it on the radio so, I knew if is very important.

RESPONDENT: what motivated me about the training is, when it started when ICARE came to look for us. Like me that they assigned to be going street to street to take down parent’s names of children of 6-23 months. I knew the importance but it didn’t motivate me but when the people I enlightened started calling me, they call me in some area and I go to educate them and that has really motivated me a lot.

RESPONDENT: What motivated me was, at first when we were called and they explained to us how to feed small children. I thought we were the only ones they called. After a little while, I now heard Imam making an announcement and giving explanation about it. I was surprised and said it is not a playing matter. After he explained, I also heard it on the media and after then I also got a text on my phone. So that motivated me and people we enlighten because they also get text messages. They agreed that this thing is important. It motivated them and me too 100%.

RESPONDENT: what motivated us on this thing is, all these things they asked us to eat are things we normally eat. We just do not know how to prepare them properly and its importance. If it had changed our type of food, it would have been a problem. We used to waste these foods, but we were called to order and thought the right way. That’s the truth of the matter. If it had changed the type of food we eat, it would have caused a lot of troubles."

**Religious Leaders:** Both mothers and fathers spoke about things which motivated them. See above

**Any other thoughts you would like to share?**

**Ward name: Rigachukun1**

*Note: For each of the questions, note if it was similar or different between the groups of fathers and mothers from each ward. Include any quotes that are particularly relevant*

**PERCEPTIONS**

Ques 1: How did fathers/mothers in the area describe their thoughts/opinions/likes and dislikes (**perceptions)** of the recommendations made by the program (CBOs, CHEWs, Religious leaders, phone calls/text messages, pamphlets, posters, feeding bowl, rec. dietary pattern) about feeding their children?  **Hint: look out for use of Perceptions + Exposure codes**

**What were the similarities?**

**CBOs: N/A**

**CHEWs**: both mothers and fathers expressed positive thoughts about their contacts with the CHEWs as well as their messages. Some mothers said that the visits of the CHEWs have had strong impact on their husbands’ mindsets about what kinds of food children require to be healthy. They said that the CHEWs had been a more credible source of IYCF messages to the husbands compared to when they as mothers had shared similar messages with the fathers and therefore the fathers had come to appreciate those messages more when hearing them from the CHEWs.

“MODERATOR: What is your opinion on the health workers coming to your house for advice on how to take care of your child?

RESPONDENT 5: All we will say is thank you

RESPONDENT 7: It has opened the mind of our husband that the children need varieties of food and fruits because when you tell them, they think we just want to enjoy ourselves.

RESPONDENT 2: If you tell your husband to buy fish, fruits, for children he will say you want to eat the fruit and the fish but now they know it’s to improve the child’s health.

RESPONDENT 8: I am impressed because they always call our husband to them the importance of foods to the health of the children from a certain month to another. They even come back home to tell us they were called. So that have motivated them unlike before when you tell them they will say you only want to eat it. They explained to them same way they explained to us.

MODERATOR: Is there any dislike on the coming of the health workers?

ALL RESPONDENTS: Nothing”

**Feeding bowl: N/A**

**Pamphlet**: both fathers and mothers had positive thoughts about the pamphlets. Father mentioned liking the photographs used. Others also mentioned that they liked that the pamphlets were self-explanatory, and provided life course based guidance on how to feed children ie pregnancy to young child.

“Moderator: What do you like about the pamphlet?

Respondent 7: As the photographs show how to make good use of the information gotten from the family. As the photographs show how to make good use of the information gotten from the family. And is shows how you can do all these in little or no time.

Respondent 9: Circulate these pamphlets to homes so that our wives would have it. When they have it, it will go a long way and they will even educate themselves through the pamphlets.

Respondent 2: Some young couples who don’t know what kind of foods to feed their children, this will be a guide to them.

Respondent 3: It serves as a short- cut to all information.

Respondent 10: There are parents that regardless of the awareness created either in the churches or mosques they will not be convinced. But with this, they can see it in stages how to feed from the pregnant mother to the child feeding and such pamphlets should be produced in large quantities.

Respondent 1: Everything is good about it. It is acceptable, the information is so clear for everyone to see it.

Moderator: What do you not like about this pamphlet?

Respondent 10: Nothing.

Moderator: Do you think this pamphlet is meant for people like you? Why? Or Why not?

Respondent 6: It is meant for us because we are fathers.

Respondent 4: To educate others.

Respondent 9: Also, I believe with education you will continue process. So, when I take it to my wife, she will in turn educate others.”---Fathers FGD

**Phone calls/text messages**: Both mothers and fathers had positive thoughts about the phone calls/text messages.

“Moderator: What are your opinions about receiving messages on young child feeding through your phone?

Respondent 8: They are more than one. I have to five of them. So when they are sent, we should not delete them so that we can make reference to them and know what to buy for the children.

Respondent 3: Yes, it will go a long way to serve people, plenty people at a particular given time. You can also forward it to others.”---Fathers FGD

**Posters:** Both mothers and fathers had positive thoughts about the posters. Both groups mentioned the illustrations on the posters of healthy children being attractive. Some fathers said that this made the posters easy to understand especially with the illiterate population.

“Moderator: What do you think about the poster?

Respondent 4: As much as possible, the posters should be plenty in circulation and the messages passed on the posters and even one can’t read, the photos of the father, the mother and their child will pass the message.

Respondent 7: Looking at the child, how healthy he is, it will motivate you to know more about how to feed your child.

Respondent 3: In addition, it’s a contributing factor, how father and mother contribute to the upbringing of the child.

Respondent 8: It promotes love in the family where the father and mother put their heads together to bring up these children with love and attention.

Respondent 10: When I look at this poster, my first impression is health is wealth, together we can do it.”----Fathers FGD

**Radio/TV/social media:** N/A. Only mothers mentioned their perceptions with radio/TV/social media

**Rec. Dietary pattern:** N/A only mothers mentioned this. see below

**Religious Leaders:** Both mothers and fathers expressed positive thoughts about the advice received from religious leaders. Mothers mentioned that due to the influence of the leaders, fathers have increased their support for child feeding by providing more frequently.

“MODERATOR: Is there anything you dislike about the advice given to us by the religious leader?

ALL RESPONDENTS: No

MODERATOR: What is your opinion on the leaders talk about child’s feeding during preaching as mothers?

RESPONDENT 6: We have seen huge progress because they tell our husbands what and what to be provided to their family at the right time

RESPONDENT 1: It a way forward for us as mother because they talk about it a lot. It is a huge improvement. They are not only told in the Mosque, even in gatherings they tell them what to provide for their children to build their body. They say it.

RESPONDENT 10: It a way forward because our men believe what our religious leader tell them

MODERATOR: Is there anything you dislike about the advice?

ALL RESPONDENTS: No. we are happy

MODERATOR: Is there any advice you learn when the preacher is preaching?

RESPONDENT 3: Give futher advice on how to feed the child at the right time. The mallams always enlighten us again on child feeding. Before, we live the child until he starts crying before we give them food. But after the sensitization from the mallams to always care about your child’s feeding and health, that is why we ensure to monitor their feeding.”---Mothers FGD

**What were the differences?**

**CBOs:** Only fathers spoke about their perceptions regarding participation in CBOs. Some fathers said that prior to participation they didn’t know what kinds of foods were affordable but healthy in feeding their children and now they do.

“Moderator: What did they recommend? What is your opinion of those recommendations? (Probe). What the liked and didn’t like.

Respondent 9: We give them advice on vital information on what to feed their children especially what will boost their brains and help their bodies build immunity against diseases.

Moderator: What new information did you learn about child feeding during the meetings?

Respondent 10: Honestly, then, before we started these meetings, I never understood what kinds of food to feed a child with ease and less expensive not until this sensitization and every one can do it. With a 100# or 200# one can provide for his child food that could nourish the child’s body. I also passed the knowledge to other people too.

Moderator: What is your opinion about having someone talk with fathers about child feeding during CBO meetings? (Probe) on what they liked and didn’t like.

Respondent 7: In my opinion, he has done something good, and what we are expected to do so that when one is educated, he in turn educates others in the community and to have an educated community entirely. As it is expected of us and as bestowed on us by God our families, in seeing how we take good care of them by providing the basic and necessary things they need in other to have a good and even better healthy life.”---Fathers FGD

**CHEWs**: N/A

**Feeding bowl:** only mothers mentioned positive perceptions of the feeding bowl. Some mothers said they appreciated the graduations in the bowl which helped them know the quantity of food to feed their children.

“MODERATOR: What did you like about the plate?

RESPONDENT 10: The quantity of food to be given is indicated in the plates and it excites me. The child will ask is this fish, meat. It makes them happy.

RESPONDENT 2: I like the plate if there is more please I need one again. Mine is broken.

RESPONDENT 8: My baby only eat food in the plate. She calls the plate fish, meat and egg.

MODERATOR: Did you dislike the plate?

ALL RESPONDENTS: Nothing because it doesn’t break.

RESPONDENT 10: They didn’t make the plates well but mine is broken”---Mothers FGD

**Pamphlet**: N/A. Both mothers and fathers had similar positive perceptions about the pamphlets. See above

**Phone calls/text messages**: No differences in perceptions mentioned

**Posters:** N/A. No differences. See above**.**

**Radio/TV/social media:** Mothers expressed positive thoughts with the radio/TV/social media advertisements and how useful the messages were. Some mothers said they specifically liked the music which played before the messages came on.

**“**MODERATOR: What did you like about these advices on tv and radio?

RESPONDENT 5: Nothing

RESPONDENT 7: Nothing we are happy, they should continue showing us.

RESPONDENT: it is for the improvement of those that those that have given birth, nursing mothers and those that are yet to give birth.

RESPONDENT 6: Music played before the program on tv. When my children hear it, they call out to me and start dancing.

RESPONDENT: all they have said is good, it cannot be thrown away. We love everything they have said. We are grateful.

MODERATOR: What did you dislike about these advices?

RESPONDENTS: Nothing. We are happy.

MODERATOR: Have you change the way you feed your children base on the advice given?

RESPONDENT 1: Yes my baby is looking strong and healthy

RESPONDENTS: We saw lot of changes in our children. They ALWAYS ask what I give him and I tell them the food we were asked to give them to improve their health.”----Mothers FGD

**Rec. Dietary pattern:** Mothers spoke positively about the diet. They said that it would not be difficult for children to eat because it would be easy to chew.

“MODERATOR: What did you think about this advice?

RESPONDENT 7: It will not be difficult for the baby to eat. It will be easy while chewing.

RESPONDENT 8: It easy for the baby to eat

MODERATOR: Have you ever tried this advice?

ALL RESPONDENTS: Yes

MODERATOR: Why did you try this advice?

RESPONDENT 2: Because we know it will be sweet for the baby

RESPONDENT1: For the baby to enjoy eating the food”---Mothers FGD

**Religious leaders:** N/A No differences observed.

**EXPERIENCES**

Ques 2: What were fathers/mothers’ thoughts on the things which worked/didn’t work **(experiences**) and the reasons raised for trying/not trying the recommendations made by the program (CBOs, CHEWs, Religious leaders, phone calls/text messages, pamphlets, posters, feeding bowl, rec. dietary pattern)? **Hint: look out for use of Experiences + Exposure codes**

**What were the similarities?**

**CBOs: N/A**

**CHEWs:** Both mothers and fathers had positive experiences with the CHEWs. Some mothers mentioned seeing differences with regards to how frequently their children fall sick (immunity) and others also said there are differences in their feeding practices because they did not give eggs, meat, fish and fruit to their children but they are doing so now.

“MODERATOR: What new experience did you learn from them?

RESPONDENT: We have gotten a lot of new things because the children we had before now were not treated the same way. By getting all these advises now, the children we are currently nursing are different in immunity from the ones from before.

RESPONDENT 8: The fruits we give to children, is new development because we don’t give fruit, fish, meat or egg before the advice.” Mothers FGD

**Feeding bowl:** N/A. Only fathers spoke about this. See below

**Pamphlets**: N/A. No experiences mentioned by fathers and mothers from this LGA

**Phone calls/text messages:** N/A. No experiences mentioned by fathers and mothers from this LGA

**Posters:** N/A. No experiences mentioned by fathers and mothers from this LGA

**Radio/TV/social media:** N/A. No experiences mentioned by fathers and mothers from this LGA

**Rec. Dietary pattern:** N/A. No experiences mentioned by fathers and mothers from this LGA

**Religious Leaders:** Both mothers and fathers expressed general positive experiences about implementing the advice received from the religious leaders.

“MODERATOR: What will you say after trying the advice on your children?

RESPONDENT: we have nothing to say other than to give thanks.

RESPONDENT: We just have to tell them that may God reward them with blessings and we experience improvements.”---Mothers FGD

**What were the differences?**

**CBOs: N/A**

**CHEWs:** No differences in experiences mentioned. Both groups mentioned positive experiences.

**Feeding bowl**: Fathers said that using the feeding bowl together with the mother in feeding the child improved their relationship with their children and endeared their children more to them

“What are your experiences with trying to apply recommendations in your families? (Probe) on what works and did and add.

Respondent 3: Myself, too I used the plate given to many child to serve given to my child to serve him. Food and also I measure up and sees the difference.

Respondent 6: Actually, after the messages, we got to the extent of saying it and practicing it and off course it will bring the child closer to you as a father. As he sees you, he comes to you and becomes closer to you.” ----Fathers FGD

**Pamphlets**: N/A. No experiences mentioned by fathers and mothers from this LGA

**Phone calls/text messages:** N/A. No experiences mentioned by fathers and mothers from this LGA

**Posters:** N/A. No experiences mentioned by fathers and mothers from this LGA

**Radio/TV/social media:** N/A. No experiences mentioned by fathers and mothers from this LGA

**Rec. Dietary pattern:** N/A. No experiences mentioned by fathers and mothers from this LGA

**Religious Leaders:** N/A both had positive experiences

**MOTIVATIONS**

Ques 3: How did fathers/mothers describe what motivated them to apply the recommendations from the program (CBOs, CHEWs, Religious leaders, phone calls/text messages, pamphlets, posters, feeding bowl, rec. dietary pattern)? **Hint: look out for use of Motivations + Exposure codes**

**What were the similarities?**

**CBOs: N/A**

**CHEWs:** both fathers and mothers said that they had been positively motivated by the improvements in the general health and wellbeing of their children to continue following the advice of the CHEWs. Some mothers said that they had seen differences in their current children compared to their previous children when they implemented the recommendations of the CHEWs. Other fathers also said that their motivations had been because of the savings they were making financially due to the reduced hospital visits of their children. They said that their children were less frequently ill as a result of practicing the recommendations by the CHEWs and therefore this motivated to keep practicing them.

“Moderator: What motivated you to apply the recommendations when feeding your child?

Respondent 8: Just as I earlier said, every father would like to see his child grow up strong and healthy without any form of little diseases that would weigh that child down.

Respondent 4: What really gives us conscience and appreciation is that you discover that you spend less money in the hospital these days. On these infants because your advice to eradicate infant mortality, infant sickness because they have been on good diet.”---Fathers FGD

“MODERATOR: What give you the courage to try the advice?

RESPONDENT (): To improve the health of the child because the physical appearances need to be improved. What you do not know is what was brought to you and asked to try it. Because you want your children to be healthy, you will try it. Before you will see children with big stomach we keep giving them carbohydrates. An advice has been brought for you and you are already in need of it. You will see changes from your previous kids. We have seen progress and hope to continue to use the advice. “Mothers FGD

**Feeding bowl:** N/A. No motivations mentioned by fathers and mothers from this LGA

**Pamphlets**: N/A. No motivations mentioned by fathers and mothers from this LGA

**Phone calls/text messages:** only mothers spoke about their motivations from the text messages. See below

**Posters:** N/A. No motivations mentioned by fathers and mothers from this LGA

**Radio/TV/social media:** N/A. No motivations mentioned by fathers and mothers from this LGA

**Rec. Dietary pattern:** N/A. only mothers mentioned motivations for trying out the food combinations

**Religious Leaders:** Only fathers mentioned this.

**What were the differences?**

**CBOs:** Mothers said that they were motivated by the changes they saw in their children when they started applying the IYCF recommendations from the CBO meetings.

“Moderator: What motivated you to apply the recommendations when feeding your young child?

Respondent 1: The motivation is from where we came and was told the benefits and we tested it and we see first- hand. I remember my little child before now she was fed with water aside breast milk, but we were asked to stop and practice exclusive breastfeeding. After six months we saw great changes in the baby”---Mothers FGD

**CHEWs:** N/A. No differences in motivations mentioned by mothers and fathers

**Feeding bowl**: N/A. No motivations mentioned by fathers and mothers from this LGA

**Pamphlets**: N/A. No motivations mentioned by fathers and mothers from this LGA

**Phone calls/text messages:** Mothers mentioned being motivated by the improved immunity of their children when they heeded to the advice

**“**MODERATOR: what encouraged you to try these advises on your children?

RESPONDENT: what encouraged us is, the child I had prior to now used to fall sick a lot and the doctor said I should change the foods i give him to eat and when I did, I experienced a lot of change because he stopped falling sick without even taking drugs. When I tried it with the children after him, I experienced a lot of change that was why they mentioned it now, I embraced it.”---Mothers FGD

**Posters:** N/A. No motivations mentioned by fathers and mothers from this LGA

**Radio/TV/social media:** N/A. No motivations mentioned by fathers and mothers from this LGA

**Rec. Dietary pattern:** Mothers said that the fact that the combination of foods would be pleasant to the baby and likely welcomed, this was their motivation for trying out those recommendations.

**“**MODERATOR: Why did you try this advice?

RESPONDENT 2: Because we know it will be sweet for the baby

RESPONDENT1: For the baby to enjoy eating the food”---Mothers FGD

**Religious Leaders:** Some of the fathers spoke about the fact that the low cost of the items spoken about by the religious leaders as being important in feeding their children was a good motivation**.**

“Moderator: What motivated you to apply the recommendations when feeding your child?

Respondent 9: The easy part of feeding of my family is that it is not as cost as I was expecting it to be initially.

Respondent 2: Also, everyman will like to see his children grow strong.

Respondent 4: What motivated me was; when you are thinking it was an expensive venture but having gone through the seminar, having gotten these advices from the Pastor we discover that it is not expensive. With a little amount, a child can get what he wants to eat and feed well.”----Fathers FGD

**Any other thoughts you would like to share?**

**Ward name: Rigachukun2**

*Note: For each of the questions, note if it was similar or different between the groups of fathers and mothers from each ward. Include any quotes that are particularly relevant*

**PERCEPTIONS**

Ques 1: How did fathers/mothers in the area describe their thoughts/opinions/likes and dislikes (**perceptions)** of the recommendations made by the program (CBOs, CHEWs, Religious leaders, phone calls/text messages, pamphlets, posters, feeding bowl, rec. dietary pattern) about feeding their children?  **Hint: look out for use of Perceptions + Exposure codes**

**What were the similarities?**

**CBOs:** N/A. Only fathers spoke about this.

**CHEWs**: Both mothers and fathers had positive thoughts on the IYCF advice received from the CHEWs. They were some differences between what mothers said and fathers said. See below

**Feeding bowl:** N/A. Only mothers mentioned this.

**Pamphlet**: Both mothers and fathers expressed positive thoughts about the pamphlets and the messages on them. Mothers mentioned liking the pictures used and that the words used are easy to understand.

“Moderator: What do you like about this pamphlet?

Respondent 3: There's nothing we don't like about it, everything in it has meaning.

Moderator: What do you not like about this pamphlet?

All Respondents: All said nothing.

Moderator: How, if at all, has this influenced how you feed your 6-23month old?

Respondent 9: Not just seeing the pictures, there are pictures with write-ups to read and understand what the picture is saying.”---Mothers

**Phone calls/text messages**: N/A. Only fathers mentioned this

**Posters:** Both fathers and mothers had positive thoughts about the posters. There were some differences though. See below.

**Radio/TV/social media: N/A.** Only mothers mentioned this.

**Rec. Dietary pattern:** N/A. Only mothers mentioned this. See below.

**Religious Leaders:** N/A. Only mothers mentioned their thoughts about this.

**What were the differences?**

**CBOs:** Fathers spoke positively about hearing other people tell them about child feeding during their meetings.

**“**Moderator: What is your opinion about having someone talk with fathers about child feeding during CBO meetings? (Probe) on what they liked and didn’t like.

Respondent 9: We will be grateful to have people who will come and educate us with our wives on issues like this and on antenatal and their likes because it is good for one to be enlightened in all he does.

Respondent 10: It’s delighting to have somebody from elsewhere to come and impact positively to your life. It doesn’t come easy though that is what you people have done for us and we show our heartfelt appreciations. So we had some persons who recommended the use of soybeans milk to us. They taught us how to extract the milk and to equally make the cake. It was nutritious to the body especially our children.it was well received and the public really appreciated it.”----Fathers FGD

**CHEWs**: Fathers said that they would prefer the CHEWs speaking to people in a language that was familiar to them but otherwise the messages were good. Although all mothers were appreciative about the advice they received from the CHEWs, some mothers said they had been initially hostile about the CHEWs visiting their homes.

“Moderator: What is your opinion of those recommendations? On what they liked and what they didn’t like.

Respondent 9: It is good to encourage them especially that they do their best to tell us what to do even in our families and they move to villages to educate those people who do not have the opportunity of coming to urban centers to listen to them speak.

Respondent 1: in my opinion, what they do is good, they also need support so that their work can be successful.

Respondent 10: In my opinion, they are doing a great job trying to see that they create awareness but I would want them to do two things; to educate people in the language commonly understood for better comprehension, secondly, if only they do the former then the people will be duly educated and their aim achieved.”---Fathers FGD

“Moderator: how was their visit? We said they came how was their visit?

Respondent 1: They came, when they came in, they greeted us and we all sat together and played with the children, we usually get angry when they came, we are now friends, they visit and we also visit them, we are happy with their coming. And they always visit us. When we have issue or not, they still come and counsel us.

Respondent 2: we are happy with their coming, they sometimes meet me or my husband, and when I’m back home, he tells me my friends were here. We are happy with their advices and God bless them all.

Respondent 8: they always call me through the phone, one of them called me three times during the corona lockdown, she will always ask if I do adhere to those advices given to me, she will ask of my child’s growth, we interact a lot with her, even if I don’t see her around, I use those advices she gave me.”---Mothers FGD

**Feeding bowl:** All the mothers had positive thoughts about the feeding bowl. Many mothers found the bowl to be attractive. They also spoke about how the pictures of foods in them were attractive and alluring to children.

**“**Moderator: What do you like about the bowl?

Respondent 5: The bowl is attractive. Before you even serve the child food, the plate will attract you.

Respondent 8: My child likes the bowl because of the pictures of the different kind of foods, he always looks at the bowl while eating. He eats the food and sees the plate as something to play with.

Respondent 7: The bowl is attractive even as an adult, it attracts you. Children like it when you put their food inside.

Respondent 1: The bowl is attractive and should be kept clean because you will like to keep well so that it doesn’t get missing.

Moderator: What do you not like about this bowl?

All Respondents: Nothing”---Mothers FGD

**Pamphlet**: N/A. Both spoke positively about the pamphlets.

**Phone calls/text messages**: Although fathers had general positive thoughts about the phone calls. Some fathers mentioning preferring text messages.

“Moderator: What are your opinions about receiving messages on young child feeding through your phone?

Respondent 6: In my opinion, I would really want to receive a text message.

Respondent 10: To me, if the government would implement a mandatory policy that would have parents to feed their children with sort of food we have been discussing here. Also, in schools, sensitization seminars should be organized. Lastly, the government should review the constitution and make it a law that “exclusive breastfeeding should be practiced”.---Fathers FGD

**Posters:** Although both fathers and mothers said positive things about the posters, fathers mentioned that the messages may not be easily seen and read by everyone and so the photographs were a plus for them. Others also said they liked the illustrations of healthy children used in the photographs.

**“**Moderator: What do you like about the poster?

Respondent 2: What we have seen on this poster we really like it and appreciate it and by the grace of God we will adhere to the message that is passed on to us through the poster.

Respondent 7: Actually, not everyone will see and be able to read the content of the poster. But the photographs there are clear and are capable of passing the message to whoever that sees it.

Respondent 10: I like the way the child looks. It shows that the child is looking healthy and well nourished.

Moderator: what do you not like about this poster?

All Respondent: There is nothing that we do not like on the poster. The poster is perfect.

Moderator: Do you think this poster is meant for people like you?

All Respondent: this poster is made or printed for everybody because it passes the message to everyone who looks at it and is feeding his or her child. It doesn’t change anything, it is our food in the rural area, and it shows how to prepare it.”----Fathers FGD

**Radio/TV/social media:** mothers mentioned general positive thoughts about the TV IYCF advertisements

“Moderator: What do you like about this information?

Respondent: In the television or radio?

Moderator: yes television, radio or through text messages.

Respondent 7: In the television, when they want to show, they show the picture of a yam.

Whatever should be given to the child is shown pictures of maize, vegetables etc.

Moderator: What do you not like about the information?

Respondent: We all like everything.”---Mothers FGD

**Rec. Dietary pattern:** Mothers mentioned that the recommended foods such as fish and eggs were tolerable to children because they were easy to chew.

“Moderator: What do you think about this advice?

Respondent: what we think about this advice is because the child will not be able to chew the food that is why they recommended this.

Respondent: you see egg is very soft. Even if a child doesn’t have teeth, the child can chew the Yolk. And they recommended fish too because the child doesn’t have teeth to chew the fish at that stage. That is why it was recommended to give a child soft food so the child can find it easy.”---Mothers

**Religious leaders:** Mothers mentioned general positive thoughts about the messages from the religious leaders. They said that the respected position of the religious leaders made fathers listen to them. They however said that they wanted the religious leaders to do this more frequently similar to how frequently the CHEWs were doing it.

“Moderator: what is the opinion about having religious leaders talk about child feeding during their sermons?

Respondent 5: I’m very happy because when he refuses to listen to the CHEW, he will listen to the Imam because they respect the Imam a lot. Involving religious leaders to announce this is good.

Moderator: what do you like and didn’t like about their recommendation from the religious leaders.

Respondent 4: what we need is for them to advice the men more just like what the CHEWS are doing. They take it seriously when it comes from the religious leaders. If you accept it and your husband accepts it, it makes you two treat it as important.

Respondent 6: the advice they gave is what our husbands should take.

Moderator: what you like and didn’t like?

Respondent 6: all the advices they gave, there is nothing I don’t like, everything is right.

Respondent 9: there is nothing that is not right, all the recommendations are ok and we are grateful. God bless them.”---Mothers FGD

**EXPERIENCES**

Ques 2: What were fathers/mothers’ thoughts on the things which worked/didn’t work **(experiences**) and the reasons raised for trying/not trying the recommendations made by the program (CBOs, CHEWs, Religious leaders, phone calls/text messages, pamphlets, posters, feeding bowl, rec. dietary pattern)? **Hint: look out for use of Experiences + Exposure codes**

**What were the similarities?**

**CBOs:** N/A. Only fathers spoke of this.

**CHEWs:** Both mothers and fathers said they had had positive experiences implementing the recommendations from the CHEWs. Mothers particularly mentioned the immunity benefits they had experienced when they practiced exclusive breastfeeding with their children compared to previous benefits. They also said that they had learnt strategies to encourage their children to eat more foods which are diverse. Some mothers mentioned seeing obvious change of character in fathers with regards to purchasing of specific food items more frequently

“Moderator: what are your experiences with applying the advice you received about child feeding?

Respondent 1: we are very grateful for these recommendations, my first children were not breastfed exclusively but because of this recommendation, I tried it on my child now. I also recommend it to others. They heard it but never tried it because they have not seen anyone applying it but now that they have seen it on my child, they are happy to apply it.

Respondent 7: thank God for this recommendation. This is my first child and I did the exclusive breastfeeding from birth to 6 month and he has never fallen ill at that time.

Respondent 8: I have seen a lot of progress, she is my first child, and there is a difference when you practice exclusive breastfeeding. There is no need to give the child water at birth before the breast milk. And about this variety of food, give the child varieties of food until the child accept all. Do not feed him one food. And do not think because a child rejected a particular food at first you will not give him again. We have tried it and seen progress. We are grateful.

Respondent 2: Thank God we’ve seen progress for these recommendations, I have applied it and seen its importance, all these illnesses my child does not do them. God bless this organization.”---Mothers FGD

“Respondent 3: we used to face difficulties but now with the arrival of CHEWs, showing us what to do, we no longer face these challenges.

Respondent 8: when I told my husband about what the CHEWS said, he said but when they met him at the mosque, he accepted the recommendations and now he has changed. He buys food for the child now. When I told my husband what the chews said about the types of food we should feed our children he said he will not be able to do it and who are the people that even said it? So the next day he came to tell me that they came to the congress to sensitize him. He didn’t understand it when I told him but when the explained to him, he understood. And I have honestly see changes. Even if I do not tell him, when he is coming back home. He brings what the child needs.”---Mothers FGD

**Feeding bowl:** None mentioned for both fathers and mothers in this area.

**Pamphlets**: None mentioned for both fathers and mothers in this area

**Phone calls/text messages:** N/A. None mentioned.

**Posters:** None mentioned for both fathers and mothers in this area

**Radio/TV/social media.** N/A. Only mothers mentioned this.

**Rec. Dietary pattern:** None mentioned by mothers and fathers in this area

**Religious Leaders:** N/A. Only mothers mentioned this. See below.

**What were the differences?**

**CBOs:** Fathers spoke about general positive experiences with applying the advice

**“**Moderator: What are your experiences with trying to apply recommendations in your families? (Probe) on what works and didn’t add.

Respondent 8: We are happy with the recommendations and the awareness brought to us by your team and our leaders we are very delighted and grateful to have you among us and the work you are doing.

Respondent 9: We are delighted with this company program on creating awareness to us and our families, we really thank you and I wish there would be women who will be able to visit our homes to enlighten our wives back there at home. Some men may be opportune to listen to such messages but because they don’t have the means to provide for their families they kept the messages to themselves. So, women should go to our homes to educate our wives will be a welcoming idea. ----Fathers FGD

**CHEWs:** None differences. Both mothers and fathers had positive experiences

**Feeding bowl**: None mentioned for both fathers and mothers in this area.

**Pamphlets**: None mentioned for both fathers and mothers in this area

**Phone calls/text messages:** None mentioned.

**Posters:** None mentioned for both fathers and mothers in this area

**Radio/TV/social media:** Mothers mentioned general positive experiences with their children when implementing the messages.

**“**Moderator: Have you made any changes in how to feed your young child as a result of this information?

Respondent 10: There's a lot of changes, we've seen Changes on our children when they eat the types of food that was asked of us to build their body.

Respondent 8: we have seen a lot of changes with messages coming in than when messages weren’t coming in. since when the messages started coming and we started using them, we've seen changes.”---Mothers FGD

**Rec. Dietary pattern:** None mentioned by mothers and fathers in this area

**Religious Leaders:** Some mothers said that fathers were being more intentional about providing food for their families. Other mothers also mentioned general positive health benefits they had seen in the appearance of their children.

**“**Moderator: what do they recommend?

Respondent 6: the advice is good. Like how we were enlightened, it makes my husband to put in more effort in buying food for the family. It is always preached in our religion to give our children food that will make them healthy. It was said on speaker at the mosque that every husband should take care of his family.

Respondent 2: when it was recommended to my husband, the way he purchased food items for me now is far better. I now cook delicious food. I have progressed.

All Respondents: all laughed.

Moderator: thank you

Respondent 2: there is progress.

Moderator: ok, thank you.”---Mothers FGD

“Moderator: what new information about child feeding did you learn from the sermons?

Respondent 10: we’ve seen a lot of changes in the children’s growth in their skin, and hair. We are grateful.

Moderator: What are your experience with trying to apply the recommendations in your families?

Respondent 7: All we have to say is thank you and may God reward you with blessings for all the recommendations we received, when you try them and see it works, then just continue with it

Moderator: The recommendations given to you, what worked and what didn't work and why they did or didn't try the recommendations

Respondent 5: God helped us, we tried all the recommendations and it worked. With little money you can achieve it to help the Children and ourselves. When you try the recommendations, your child will be big and healthy compared to other child that has not experienced it the child will have skinny will have skinny leg and a big stomach because he/she has not been fed well while your own child will be attractive and people will like to carry him.”---Mothers FGD

**MOTIVATIONS**

Ques 3: How did fathers/mothers describe what motivated them to apply the recommendations from the program (CBOs, CHEWs, Religious leaders, phone calls/text messages, pamphlets, posters, feeding bowl, rec. dietary pattern)? **Hint: look out for use of Motivations + Exposure codes**

**What were the similarities?**

**CBOs:** None mentioned for fathers and mothers

**CHEWs:** N/A. Only mothers mentioned this

**Feeding bowl:** None mentioned for both fathers and mothers in this area

**Pamphlets**: None mentioned for both fathers and mothers in this area

**Phone calls/text messages:** None mentioned for both fathers and mothers in this area

**Posters:** None mentioned for both fathers and mothers in this area

**Radio/TV/social media:** None mentioned for mothers and fathers in this community

**Rec. Dietary pattern:** N/A. Only mothers mentioned this.

**Religious Leaders:** N/A. Only mothers mentioned this.

**What were the differences?**

**CBOs:** None mentioned for fathers and mothers

**CHEWs:** Mothers mentioned that the progress they saw when they started applying the recommendations in turn motivated them to continue trying them. Others also said that when they realized that the foods recommended were not costly, it was motivation to try them. Other mothers also said that the persistence of the CHEWs made them believe whatever they were saying was important and so they decided to try the recommendations.

“Moderator: what motivated you to apply the recommendations when feeding your child? What motivated you to apply this?

Respondent 4: what motivated me to apply this recommendation was when I tried it and saw its positive effect. I saw a lot of progress

Respondent 5: what motivated me to apply all these recommendations was that, all these foods we were asked to feed our children with are not foods that are expensive. With little money, we can get these things. You must not have plenty money to buy them, that was our thinking. We are so happy now, with little money, we can achieve it.

Respondent 8: when I saw them coming every time, I never took them seriously. I sat down one day and thought of it that it must be very important for them to come always. I have accepted it whole heartedly now and have seen its benefits thank you so much.”---Mothers FGD

**Feeding bowl**: None mentioned for both fathers and mothers in this area

**Pamphlets**: None mentioned for both fathers and mothers in this area

**Phone calls/text messages:** None mentioned for both fathers and mothers in this area

**Posters:** None mentioned for both fathers and mothers in this area

**Radio/TV/social media:** None mentioned for mothers and fathers in this community

**Rec. Dietary pattern:** Some mothers mentioned the persistence of the CHEWs visits to their home as motivation to try the recommendations. Others also said that their rival wives were trying them and were seeing results and so that motivated them to try the recommendations as well.

**““**Moderator: Why have you tried?

Respondent 8: I tried it because the CHEWs are always coming to my house and even in the mosque. I now made up my mind that even if I do not practice it, I must try it. And I tried it and saw its importance.

Respondent 3: I tried the recommendation because I stayed with my co-wife when she applied the recommendations. We were at home when an organization came and weighed her child, they were weighing children that are currently on exclusive breastfeeding and the ones that were not practicing it and the child won. She was given #3000 that was what prompted me to also try it.”---Mothers FGD

**Religious Leaders:** Some mothers said that hearing from the religious leaders motivated them to start applying the recommendations speaking to the respected position of these leaders in the community. Others also said that when they started applying the recommendations, they saw a difference in their children’s health and immunity and therefore this motivated them to continue.

**“**Moderator: What motivated you to apply the recommendations when feeding your child?

Respondent 4: When they came with these recommendations, I refused to accept it but when I later tried it, I saw a big difference with my child's health that was what motivated me to continue. Thank God he has prevented sickness from us.

Respondent 9: Going for immunization at the hospital, we were informed there and we are visited at home and also heard from the religious leaders that was what encouraged me to start.”----Mothers FGD

**Any other thoughts you would like to share?**

**Ward name: Rigasa 1**

*Note: For each of the questions, note if it was similar or different between the groups of fathers and mothers from each ward. Include any quotes that are particularly relevant*

**PERCEPTIONS**

Ques 1: How did fathers/mothers in the area describe their thoughts/opinions/likes and dislikes (**perceptions)** of the recommendations made by the program (CBOs, CHEWs, Religious leaders, phone calls/text messages, pamphlets, posters, feeding bowl, rec. dietary pattern) about feeding their children?  **Hint: look out for use of Perceptions + Exposure codes**

**What were the similarities?**

**CBOs:** N/A

**CHEWs**: Both mothers and fathers spoke positively about their thoughts about the advice received from the CHEWs. Mothers mentioned that the CHEWs were teaching them what actions to take to care for their children

“Moderator: What is your opinion about having CHEWS talk about young child feeding during their visits to your home? (Probe) on what they liked and didn’t like.

Respondent 10: We are welcoming the idea and recommendations and we are giving them our support. That is our opinion” ---------Fathers FGD

“MODERATOR: what do you think about this advice?

RESPONDENT 9: they are trying to show us how to care for our children by giving them adequate nutritious food that gives strength and good health.

RESPONDENT 6: the advice they give us will help us a lot.” --------Mothers FGD

**Feeding bowl:** N/A

**Pamphlet**: Both mothers and fathers report similar things about their thoughts, likes, etc about the pamphlets. Both groups mentioned that they liked everything about the pamphlets. The fathers group mentioned the images in the pamphlets are easy to understand.

“Moderator: What do you like about the Pamphlet?

Respondent 8: What fascinates me with this pamphlet is the position the mother takes in feeding her child. Also, the care a woman receives while pregnant.

Respondent 7: What I like about the pamphlet is the sequential arrangement of the images such that any one that picks it will know the stages such that any one that picks it will know the stages involved.

Moderator: What do you not like about this pamphlet?

Respondents: Nothing at all (collectively).

Moderator: Do you think this pamphlet is meant for people like you? Why? or Why not?

Respondents: Yes (collective response)

Respondent 4: It is meant for people like us and we will in turn reach out to those who are not here.

Moderator: What do you think about this advice?

Respondent 2: This is a good advice and when we follow the advice, we will benefit a lot from it.

Respondent 4: It is accepted in our community and people like me who are in tune with it” Fathers FGD

**Phone calls/text messages**: N/A

**Posters:** Both groups spoke positively about their thoughts on the posters. Both groups were positive in their descriptions of what they liked about the posters. Some mothers mentioned how the variety of foods displayed on the posters were attractive. Other mothers also talked about how attractive the healthy children displayed on the posters were. Some fathers also mentioned how “fascinating” it was that the father in the poster was sitting down to feed the child. Other fathers also mentioned how they appreciated that the suggested foods in the posters were available locally and inexpensive and therefore this will be easy to adopt.

“MODERATOR: What do you like about the poster?

RESPONDENT 1: It encourages me to do more and prepare what to give the child.

RESPONDENT 4: We've seen the poster and was motivated to do more and also to strengthen our child and help them build their body.

MODERATOR: Why do you like this poster?

RESPONDENT 10: Because of the things on the poster, it’s important when you read what is on the poster.

RESPONDENT 2: When you read what is on the poster, you'll know how important it is to use those things for your child.

RESPONDENT 3: When you see that child on the poster very attractive, you would want to see your own child look like that” Mothers FGD

**Rec. Dietary pattern:** N/A Only mothers mentioned their perceptions regarding this.

**Radio/TV/social media:** None mentioned for mothers and fathers in this community.

**Rec. Dietary pattern:** N/A. Only mothers reported this.

**Religious leaders: B**oth mothers and fathers spoke positively about the recommendations from the religious leaders. Some mothers said that some fathers would not listen to their advice but would be more willing to listen to the religious leaders and so they were appreciative of the religious leaders speaking about IYCF.

**“**MODERATOR: What is your opinion about having religious leaders talk about child feeding during the sermon?

RESPONDENT 9: We appreciate them for their contribution on this matter, Thank you for the information.

MODERATOR- What do you like about religious leaders preaching about this and what you didn't like?

RESPONDENT 5: Some of the men don't usually listen to their wives but sharing the information with them at the mosques makes them accept it.

RESPONDENT 3: They accept it when they hear it from the mosque. They disagree with the wife when she tells him but from the mosques, makes them apply it.

MODERATOR: What new information about child feeding did you learn from the sermon?

RESPONDENT 7: we've heard a lot of things that were useful, most women don't care for their children, they leave the child without food but with this information preached and shared with us on the time to feed children, we have applied it and we are happy”---Mothers FGD

**What were the differences?**

**CBOs:** Mothers did not report perceptions with CBOs. Only fathers did.

“Moderator: What is your opinion about having someone talk with fathers about child feeding during a community-based organization meeting?

Respondent 2: It is good because it exposes us to things that we suppose to know about child feeding. It is good because every day in life you will get to find out about something you have but you do not know their importance until someone comes to enlighten you about it. It might be a tree in your house that you just use as shade but someone will come and tell you its importance and you will see that it is true. So, the different organizations that come to enlighten the public on these types of things are of great importance and the general public is supposed to accept them with both hands so as to gain more knowledge, improve their lives and prevent diseases from getting to small children in other to save money and improve.” Fathers FGD

**CHEWs**: Some fathers mentioned that they would prefer the CHEWs to schedule visits ahead of time or visit them in their community groups
“Respondent 4: My opinion is that if possible, let them fix a visit schedule that they would be coming to visit us and create this awareness to our families.

Respondent 3: If they had the opportunity, they can visit us and even our groups and others that we associate with, we will like them to come and create this awareness to us” ------Fathers FGD

**Feeding bowl:** Fathers did not report perceptions to feeding bowl. Only mothers did

“Moderator: How do you use this plate?

Respondent 2: A child of 6 month should be given food up to red line and at 11 month we should give food at minimum green line while above 23 month we give our baby food that will fill the plate to eat.

Respondent 1: A child of 6 month should be given food up to the redline while 11 month food at green and a baby at 21 month we should fill the plate.

Moderator: What did you like about the plate?

Respondent 8: The plate is good but baby can easily break it while eating.

Respondent 2: The plate can break easily because my only last for 1 month. I wish the plate is metal in nature so that whatever the child does with it, it doesn’t break.

Moderator: What did you dislike about the plate?

Respondent 2: The Plate break easily

Respondent 10: The Plate break easily

Respondent 1: The plate break easily compare to the previous once given to us that is unbreakable.

Respondent: I have something to say. The plate is very good. No matter what a child does with it, it doesn’t break compared to other places. It is very good.”

**Pamphlet**: None observed.

**Phone calls/text messages**: Mothers did not report this. Fathers were the only ones who reported perceptions concerning phone calls/text messages. Some fathers recommended that the source of the messages should be indicated in the text message. Others also spoke about preferring text messages over phone calls.

“Moderator: What are your opinion about receiving messages on young child feeding through your phone?

Respondent 4: Sending these messages is very important because it is a source of new information and a reminder as well. If possible, let every person receive the message. To me, I think sending messages will be better than the call and it will be cheaper for those sending it.

Respondent 1: We are welcoming the idea of receiving the messages because it is very important for us. We that didn’t get it are in need of it.” Fathers FGD

**Posters: N/A.** Both soke positively. See above**.**

**Radio/TV/social media:** None mentioned for mothers and fathers in this community.

**Rec. Dietary pattern:** Mothers spoke positively about the advice of the combination of foods

**“**MODERATOR: What do you think about the advice?

RESPONDENT 10: The advice was useful

RESPONDENT 6: we've tried it and seen its usefulness

MODERATOR: What do you think about this advice?

RESPONDENT 1: my thought is to add more.

MODERATOR: Add what?

RESPONDENT 1: More advice

MODERATOR: Have you tried this advice?

RESPONDENT 7: yes, we've tried it and it seen how important it is”---Mothers FGD

**Religious Leaders:** None. Both report positive thoughts.

**EXPERIENCES**

Ques 2: What were fathers/mothers’ thoughts on the things which worked/didn’t work **(experiences**) and the reasons raised for trying/not trying the recommendations made by the program (CBOs, CHEWs, Religious leaders, phone calls/text messages, pamphlets, posters, feeding bowl, rec. dietary pattern)? **Hint: look out for use of Experiences + Exposure codes**

**What were the similarities?**

**CBOs:** N/A

**CHEWs:** N/A

**Feeding bowl:** N/A

**Pamphlets**: N/A

**Phone calls/text messages:** No experiences recorded for both mothers and fathers

**Posters:** N/A. Only mothers spoke of their experiences with the posters.

**Radio/TV/social media:** None reported for mothers and fathers in this community

**Rec. Dietary pattern:** N/A. Only mothers mentioned this

**Religious Leaders:** Both mothers and father had positive experiences with applying the recommendations from the religious leaders. Some fathers mentioned seeing differences in the general health, appearance and intelligence of their current children compared to their older ones. Some mothers also mentioned observing that their children were sleeping better than before when they began applying the recommendations.

**“**Moderator: What are your experiences with trying to apply the recommendations in your families?

Respondent 1: We have tried and tested it already and we see it. For me, the child that was wined before is not as healthy, nourished and smart as this one now.”---Fathers FGD

**“**MODERATOR: What are your experiences in trying to apply the recommendations in your families?

RESPONDENT 10: We are grateful and God bless you. May God give you the grace to share more information with us.

RESPONDENT 4: God bless you; we are grateful and may God pay you back

MODERATOR: Still with the experience with trying these recommendations, what worked and didn't work, and why you did and didn't try the recommendations?

RESPONDENT 7: we've tried it and are happy with it. We should try and feed our children on time and be patience with them when eating, make sure you feed the child first before doing any of the house chores that will take your time. When the child is fed well, he sleeps well.

RESPONDENT 2: The mother should always try and prepare the child's food before the child start crying for food. If the child does not want to eat at that time, keep the food. When he is ready, give him the food to eat and be satisfied.”----Mothers FGD

**What were the differences?**

**CBOs:** Mothers did not report experiences with CBOs. Only fathers did

“Moderator: What are your experiences with trying to apply the recommendations in your families? (Probe) On what worked and didn’t work and why they didn’t try the recommendations.

Respondent 3: We will try to adhere to the advice given to us.

Respondent 4: We are glad with the pieces of advice given to us, because if we follow it, our children will have a nourished healthy and strong life.

Respondent 5: We are currently receiving the advice, and we will need more because we have practiced it have reaped the benefits except for what we hope to get ahead. But from what we have learned, we ought to give you thanks for the good work. Children have gained more weight and strength.

Respondent 10: I like this group and I think I came at the right time being that this is my first child. Since when I started receiving the message from the church, I became keen and focus. Today is my first day of coming here and I am really happy being here. I have learned a lot of things today. My intention here is that since I am lucky to be here today, with the lessons I have learned, it is my wish that I put into practice all that I have acquired today so that I can reap its benefits.

Respondent 2: This program is like a test not of faith of judgment, but similar to it in the sense that what God gave this group, they deem it fit to come and share with others. It is a good thing that you have done and we are really grateful. Anybody that have tried it will never say it doesn’t work.” Fathers FGD

**CHEWs:** fathers did not report experiences with CHEWs. Only mothers did
“MODERATOR: what worked and did not work and why did you or did not try the recommendations?

RESPONDENT 2: they always advise us to care for children, we should always be patient when we don’t have the means to buy these foods. We are seeking for help from the government.

RESPONDENT 7: we tried it and seen its usefulness because my daughter was eight months when she started walking I’m happy I tried those recommendations.

RESPONDENT 10: I have tried it, and if you have the means please do try it. Glory be to God, we seen progress.” Mothers FGD

**Feeding bowl**: Fathers did not report experiences with the feeding bowl. Only mothers did

“MODERATOR: Have you tried this advice?

RESPONDENT 7: yes, we've tried it and it seen how important it is” Mothers FGD

**Pamphlets**: Fathers did not report experiences with pamphlets. Only mothers did

“MODERATOR: How, if at all, has this influenced how you free your 6-23-month-old?

RESPONDENT 1: We are happy, we've checked and its ok for us, we are happy.

RESPONDENT 7: The way I saw the children in good health and strength that's what influenced me.

RESPONDENT 3: What attracted me was when I saw the man was playing with the child, He carried the child up and was playing with him, that's what attracted me” Mothers FGD

**Posters:** Some mothers said they noticed general healthful differences in their children now compared to their older children when they tried the recommendations of the posters.

**“**MODERATOR: Have you made any changes on how to feed your young child as a result of this information?

RESPONDENT 9: yes, there are changes

MODERATOR: What is the change?

RESPONDENT 9: Your child will be healthy compared to the ones their mothers have never tried this recommendation on them. Even you that try it will see the difference between the child and the elder siblings you didn’t try it.”--**-Mothers FGD**

**Radio/TV/social media:** None reported for mothers and fathers in this community**.**

**Rec. Dietary pattern:** Mothers spoke of general positive experiences with trying the recommended foods

“MODERATOR: Have you tried this advice?

RESPONDENT 7: yes, we've tried it and it seen how important it is”---Mothers FGD

**Religious leaders:** None. Both report positive experiences. See above

**MOTIVATIONS**

Ques 3: How did fathers/mothers describe what motivated them to apply the recommendations from the program (CBOs, CHEWs, Religious leaders, phone calls/text messages, pamphlets, posters, feeding bowl, rec. dietary pattern)? **Hint: look out for use of Motivations + Exposure codes**

**What were the similarities?**

**CBOs:** None mentioned for mothers and fathers in the community

**CHEWs:** N/A. Only mothers mentioned this

**Feeding bowl:** N/A. Only mothers mentioned this. See below.

**Pamphlets**: No motivations mentioned by fathers and mothers in this community.

**Phone calls/text messages:** No motivations mentioned by fathers and mothers in this community

**Posters:** No motivations mentioned by fathers and mothers in this community

**Radio/TV/social media:** No motivations mentioned by fathers and mothers in this community

**Rec. Dietary pattern:** N/A. Only mothers mentioned this. See below.

**Religious Leaders:** Both mothers and fathers spoke about the respectable position of the imam as the motivation to try out his IYCF recommendations compared to when they had heard similar from the CHEWs.

**“**MODERATOR: What motivated you to apply the recommendations when feeding your child?

RESPONDENT 1: We've tried it and are happy with it and that's why we continued with it

RESPONDENT 8: When the CHEWs came and shared the recommendations with us, we took them for granted not until we heard from the religious leaders, and whatever the religious leaders say is true, they don't play with their subjects, and that's what motivated US to do it.”---Mothers FGD

**What were the differences?**
**CBOs:** None mentioned for mothers and fathers in the community

**CHEWs:** Some mothers said that the CHEWs themselves had children who looked attractive and healthy and so this motivated them to try the advice the CHEWs were recommending.

“MODERATOR: what motivated you to apply the recommendations when feeding your child?

RESPONDENT 4: We were motivated and we tried it. It is very good.

MODERATOR: what motivated you? There’s something that motivated you.

RESPONDENT 1: because it is good, that was why I tried it and I’m happy for trying it.

RESPONDENT 7: because you see other children in good health and strength and your own is lacking, that was why I tried it and was motivated to try it.

RESPONDENT 9: what motivated me was the CHEW, they know what will benefit your child and the ones that will not benefit the child. When they came, I thought they were just doing our job not until I heard it on the radio and now, they followed me home with the materials, explaining it to me, that was why I tried it and I’m happy.

RESPONDENT 8: what motivated me was the children of the CHEWS. The children are always very attractive, that was why I tried it. The children are always attractive compared to other children. I will continue to try these recommendations.”---Mothers FGD

**Feeding bowl:** Some mothers said their desire to see their children healthy and strong was motivation to use the feeding bowl.

**“**MODERATOR: Why did you try this advice?

RESPONDENT 7: Why I tried this advice was because I want to see my child healthy, strong and be able to play. When you remember these recommendations, you would want to buy these foods for your child to eat and be strong and healthy.”---Mothers FGD

**Pamphlets**: No motivations mentioned by mothers and fathers in this community

**Phone calls/text messages:** No motivations mentioned by fathers and mothers in this community

**Posters:** No motivations mentioned by fathers and mothers in this community

**Radio/TV/social media:** No motivations mentioned by fathers and mothers in this community

**Rec. Dietary pattern:** Mothers their desire to see their children healthy was their motivation to try the recommended diets.

**“**MODERATOR: Why did you try this advice?

RESPONDENT 7: Why I tried this advice was because I want to see my child healthy, strong and be able to play. When you remember these recommendations, you would want to buy these foods for your child to eat and be strong and healthy.”---Mothers FGD

**Religious Leaders:** None. See above

**Any other thoughts you would like to share?**

**Ward name: Rigasa 2**

*Note: For each of the questions, note if it was similar or different between the groups of fathers and mothers from each ward. Include any quotes that are particularly relevant*

**PERCEPTIONS**

Ques 1: How did fathers/mothers in the area describe their thoughts/opinions/likes and dislikes (**perceptions)** of the recommendations made by the program (CBOs, CHEWs, Religious leaders, phone calls/text messages, pamphlets, posters, feeding bowl, rec. dietary pattern) about feeding their children?  **Hint: look out for use of Perceptions + Exposure codes**

**What were the similarities?**

**CBOs:** N/A

**CHEWs**: N/A.

**Feeding bowl:** N/A

**Pamphlet**: N/A

**Phone calls/text messages**: N/A

**Posters:** N/A. Both mothers and fathers reported positive thoughts about the posters. One of the fathers mentioned that he liked that the image of the child used in the posters was “chubby”. Another father also mentioned that the messages point to the fact that fathers “can take good care of their children”. Mothers also mentioned that the image used in the posters was of a child who “look(ed) healthy”, was “smiling” and was “chubby” was “fascinating” and pleasant to them. Other mothers also mentioned that the fact that the mother was “sleeping and breastfeeding” was good because some women “(breastfeed) their children while standing”.

“Moderator: what do you like about this poster?

Respondent 5: the child is very chubby

Respondent 1: the messages are important, so we call on groups to use such to pass their messages across to others.

Respondent 7: the picture of the child there is what I like most on the poster.

Moderator: do you think this poster is meant for you?

Respondent 6: it is meant for everybody whether those in the city or village.

Participants seeing the poster for the first time.

Moderator: what is the poster asking you to do?

Respondent 6: it shows that a father should take good care of his family and ensures that he provides what they need to eat and grow well.

Respondent 2: it is asking me to take care of the mother of the child and the child too so that they will both be healthy.”----Fathers FGD

“MODERATOR: What did you like about the poster?

Respondent 7: I just keep meditating on the poster on how to take care of my child.”---Mothers FGD

**Radio/TV/social media:** N/A

**Rec. Dietary pattern:** N/A

**Religious Leaders:**  Mothers believe that receiving advice from religious leaders is important because they are influential to the members of the community.

“MODERATOR: What is your opinion seeing religious leaders, community leaders talking on child feeding?

Respondent 9: it serves as reminder to the men and also the importance of the discussion. We like the advises they give because the things happening now wouldn’t have been happening.

Respondent 8: It serves as reminder. What she said is true. They sensitize them in the mosque always and they sensitize us in school too on how care for our children, homes and even our husbands and it makes us really happy because when a man doesn’t act right and he hears those sermons, we see a lot of changes.

Respondent 10: They will know the importance of the topic, because they believe what religious leaders say. It is good for them to keep sensitizing them because it increases the faith of a man in caring for his family and providing the foods their will increase their blood for them to add to their cooking and eat.” Mothers FGD

**What were the differences?**

**CBOs:** Mothers did not report perceptions with CBOs. Only fathers did. Fathers spoke positively about the recommendations received and said they supported them fully.

Moderator: What did they recommend? What is your opinion of those recommendations? (Probe). What the liked and didn’t like.

Respondent 2: The recommendation received from the gathering was that it is very important that we received and accepted it because it will help our children to grow well when we practice what we have been lectured on.

Respondent 5: My opinion on the recommendation is that I support the program in its totality (100%). This is because if your son is well fed and healthy, it just as good as you are being the person healthy.

**CHEWs**: Mothers did not report perceptions with CHEWs. Only fathers did. Some fathers mentioned that other family members had not been welcoming of the idea of someone else teaching them “how to feed their children” although they themselves were fine with it. Other fathers also said that CHEWs coming to their homes to talk with fathers would encourage the mothers to take the advice of the CHEWs more seriously if they werent initially doing so. Several mothers also expressed positive thoughts about the advice received from the CHEWs and the frequency of the visits. They said that the advice given was good and they liked how frequently the CHEWs came to their homes to educate them and encouraged more CHEWs to be involved in reaching more women/families at home.

“Moderator: if not, would like the CHEW to schedule visits when you are home?

Respondent 9: we really want them to come around because if they met the women and at that point they were not serious about it, when they see the father give audience and accept them, they also will give audience.” Fathers FGD

“Moderator: What is your opinion about having CHEWS talk about child feeding during their visits to your home? (Probe) on what they liked and didn’t like.

Respondent 10: Actually, it’s nice. To some, they show same level of care, but to some, they don’t. But when you express some level of hospitality to them then they feel relaxed and happy that they have your attention. It makes them happy.

Respondent 6: When they came to my house, my younger brother wasn’t happy with their presence and he’s said. “Why will they come to teach us how to feed children?” I rebuked him and then explained things to him on showing hospitality, besides that, they were doing their job.”---Fathers FGD

“MODERATOR: What do you like and dislike on the advice given by the health workers

Respondent 3: All the advice given is good and I use them.

Respondent 8: All the advice is good thank you very much, may God bless.

Respondent 9: All I will say is thank you very much, all the advises are good.

MODERATOR: What is your opinion on these advice?

Respondent 2: The advice is good my children are looking healthy and strong like foreign people’s children rather than before when they looked like poor people’s children. After they gave the advice and we practiced it, our children are now very attractive.

Respondent 3: I like the growth of my children now because she looks so beautiful and chubby and I am very happy.

MODERATOR: Is there new ideal you have learnt from the health workers.

Respondent 6: Cleanliness and type of food to be given to the baby. Because some people were very careless about it, they will not check what they give their child, if it was covered or not, they will just carry it and give to their children. But their coming made us know how to go about it.

Respondent 8: Cleanliness and constant feeding our baby. Honestly, when they first came I never knew children could be given such foods until they told me you can give them and when you give them it is very important for their health.

Respondent 1: Cleanliness. What she said is true because all they told us are important. Because if you see my daughter now, you will think she is 2 years old while is isn’t.

Respondent: Even me when I first brought my child, he was stooling. But when I tried these foods and practiced good hygiene, he is now healthy and smart. Glory be to God

MODERATOR: What is your opinion on health workers visiting your houses on child feed?

Respondent 1: they should be encouraged more to come to houses like they do.

Respondent 7: They should increase their number so that they can visit other places too.

MODERATOR: Did you like or dislike their coming

Respondent 8: We like the way they always came to our houses, they should keep coming.

Respondent 10: We like the way they always came to our houses and they should add more people so that they can reach places they haven’t.

MODERATOR: What will you say on the advices given on your family?

Respondent 3: thank you very much, May Allah will bless you all. We are very happy

Respondent 5: Thank you very much God will bless them.

Respondent 8: Thank you very much, May Allah will bless you all.”-----Mothers FGD

**Feeding bowl:** N/A. Only mothers spoke about their perceptions related to the feeding bowl. Some mothers spoke about how appreciative they were of the graduations in the feeding bowl.

“MODERATOR: what do you like about the plate?

Respondent 3: the food limit in the plate even though I do not have and have only seen it with my neighbour that was given.” ---Mothers FGD

**Pamphlet**: N/A. Only fathers spoke about their perceptions related to the pamphlets. One father mentioned how he disliked the fact that the mother in the pamphlet was standing and breastfeeding and that mothers are supposed to be seated when breastfeeding in order to position the head of the child appropriately. Another mentioned that a woman using a breast pump in the pamphlet was not acceptable to him.

“Moderator: what do you not like about the pamphlet?

Respondent 6: the way this woman is standing and breastfeeding her child is what I don’t like from the pamphlets.

Respondent 2: what this woman is doing is what I don’t like (the photo of a woman pumping breast milk)

Moderator: what do you think the woman is doing?

Respondent 8: she is milking her breasts so that she would put it in a self-administered feeder for the child, to either feed himself also else to feed him.

Moderator: what happens if she goes out to work or for a period of three hours before returning, she milks the breasts and keeps for the child?

Respondent 2: if she milks the breasts and keeps for the child, other members of the house with feed her.

Moderator: Do you think this pamphlet is meant for people like you?

All respondents: yes and for everybody.

The participants seeing the pamphlets for the first time

Moderator: what is this pamphlet asking you to do?

Respondents 2: the producers should try and translate the language in Hausa especially for those in the rural areas so that they too can read and understand

Respondent 3: it is very important for mother to breastfeed her child from birth to the first six months. It is very important for the child.

Respondent 7: the pamphlet says breastfeed the child 1 hour after birth but there are situations that the mother would have complications during birth that will not able to breastfeed within the stipulated time?

Moderator: unless otherwise the stipulated time is 1 hour

Respondent 9: concerning the feeding positions, mothers are expected to sit comfortably and positioned the head of the child well not like the one we see stand and feed the child.”---Fathers FGD

**Phone calls/text messages**: Mothers did not report perceptions with phone calls/text messages. Only fathers did. Fathers mentioned the phone calls and text messages serving as reminders for them and for their wives as well who sometimes read their messages.

“Moderator: what are your opinions about receiving messages on your phone?

Respondent 6: I will want to receive this message or messages. This is because I value the programme and the way I do my things, I I read every message on my inbox even those of the service providers (MTN).

Respondent 1: we will like to receive the message because as a human, we are bound to forget. So, the messages will serve as reminders to us when ever forget foods we are expected to feed our children. Also, our wives also pick up the phones and read these messages so that they too would be educated on that. Another thing is that some friends may pick up your phone and see the messages and they too will be enlightened.”---Fathers FGD

**Posters:** Fathers did not report perceptions with posters. Only mothers did. N/A. Both mothers and fathers reported positive things. See above.

MODERATOR: What did you like about the poster?

Respondent 7: I just keep meditating on the poster on how to take care of my child.

Respondent 2: Seeing the baby smiling at me. It fascinates me

Respondent 1: Seeing how healthy the baby is looking.

**Radio/TV/social media:** Fathers did not report perceptions with radio/TV/social media. Only fathers mothers did. Mothers mentioned that they liked that the children used in the advert were “fine children”. Others also mentioned liking how much well explained the adverts were to try to make sure that those (that) understand (them) are plenty”

“MODERATOR: Is there anything you like about the advice?

Respondent 5: The part that talked about feeding of children and the part displayed fine children.

Respondent: foods on child feeding.

Respondent: I like how they explain well while sensitizing us because if some do not understand, some will but those they understand are plenty”---Mothers FGD.

**Rec. Dietary pattern:** Mothers did not report perceptions with rec. dietary pattern. Only fathers did. Fathers said that they liked that the foods recommended are locally available and inexpensive.

Moderator: the infant and young child feeding programme in your area suggests that fathers can procure nutritious food like eggs, fish, sweet potatoes, spinach pumpkins and beans to be fed to their 6 to 23-month-old child

“Moderator: what do you think about this advice?

Respondent 5: our thought is that they are very important because most of these foods such as eggs, spinach and pumpkins is not expensive. Most times you see that pumpkins grow around our houses and people do not its importance but because of programs like this, we are more aware because we need not buy them.”---Fathers FGD

**Religious leaders:** N/A

**EXPERIENCES**

Ques 2: What were fathers/mothers’ thoughts on the things which worked/didn’t work **(experiences**) and the reasons raised for trying/not trying the recommendations made by the program (CBOs, CHEWs, Religious leaders, phone calls/text messages, pamphlets, posters, feeding bowl, rec. dietary pattern)? **Hint: look out for use of Experiences + Exposure codes**

**What were the similarities?**

**CBOs:** N/A

**CHEWs:** N/A

**Feeding bowl:** N/A

**Pamphlets**: N/A

**Phone calls/text messages:** N/A

**Posters:** N/A

**Radio/TV/social media:** N/A

**Rec. Dietary pattern:** N/A

**Religious Leaders:** N/A

**What were the differences?**

**CBOs:** Mothers did not report experiences with CBOs. Only fathers did. One father mentioned he had seen improvements in the appetite and general physical appearance of his child after implementing the recommendations from the CBOs.

Moderator: What are your experiences with trying to apply the recommendations in your families? (Probe) on what worked and didn’t work and why they did or didn’t try the recommendations.

Respondent 6: The recommendations were welcomed by me, I accepted them and I started practicing it for my child. The months earlier then I started, he didn’t start taking other foods but now I’m happy because he is eating and I have seen the changes in him. He is looking very healthy.

**CHEWs:** Only mothers reported this. Mothers mentioned experiencing positive things related to weight gain and general health of their children.

“MODERATOR: What will you say after trying these advices?

Respondent 10: we have tried these advises very well and seen that our children look different compared to the previous ones.

Respondent 3: Before man and God we have improved a lot and all we will say is thank you very much and May God bless you.

Respondent: we have really benefitted because our children’s health has changed, they are now healthy and the child will grow from an infant to a child and children’s have added weight a lot.

Respondent: what she said is true because the child’s health is different and we are really grateful and may God bless you.” ---Mothers’ FGD

**Feeding bowl**: N/A

**Pamphlets**: N/A

**Phone calls/text messages:** Mothers did not report experiences with phone calls/text messages. Only fathers did.

Moderator: what are your experiences with trying to apply the recommendations from the messages in your families? (Probe on what worked and did not work? And why they did and did not try recommendations.

Respondent 8: the organization is doing well by sending us these messages. We are also asking them to please send more because they are very useful. Sometimes, women don’t take things or information seriously. For example, the use of facemasks that was echoed and re-echoed by the government, people did not take precaution not onto doctors recommended that people should use it. That they started. Therefore, the women will respect and obey it because it is coming from a reliable source.

**Posters:** N/A

**Radio/TV/social media:** N/A. Fathers did not report any experiences with radio/TV/social media. Only mothers did. Mothers reported that they saw health and immunity benefits when they implemented the advice from the radio/TV/social media. Their children were less frequently ill over extended periods of time (1 year) particularly for children with sickle cell (SS) genotypes.

“MODERATOR: is there any changes you have effected due to the advice.

Respondent 9: For me I have a child that is SS but since I follow the advices he has not fallen sick for 1 year now.

Respondent 7: I am happy because all the advices are good.

Respondent 3: My baby always feels sick but since I stick to the advice she is very okay.”---Mothers FGD

**Rec. Dietary pattern:** N/A. Fathers did not report experiences with the recommended dietary pattern. Only mothers did. Mothers reported positive experiences with implementing the pattern and that it “makes children healthy and strong”

“MODERATOR: have you ever tried these advices?

Respondent 10: Yes I have tried it because it makes children health and strong.”---Mothers FGD

**Religious Leaders:** N/A.

**MOTIVATIONS**

Ques 3: How did fathers/mothers describe what motivated them to apply the recommendations from the program (CBOs, CHEWs, Religious leaders, phone calls/text messages, pamphlets, posters, feeding bowl, rec. dietary pattern)? **Hint: look out for use of Motivations + Exposure codes**

**What were the similarities?**

**CBOs:** N/A

**CHEWs:** N/A. Both mothers and fathers reported positive things which motivated them to try out the recommendations of the CHEWs. Some fathers spoke about how they realized that the recommendations made sense logically, while others said that they felt the recommendations did not seem “harmful” but rather beneficial. Other fathers also spoke about being motivated by a young child in the community whose appearance was “chubby and strong” and who was among the children they had seen being “fed with such foods” as recommended by the CHEWs. Some mothers also spoke about the readiness of fathers to purchase the foods recommended by the CHEWs as their motivation. They also mentioned that seeing initial positive results further motivated them to continue implementing the recommendations.

“Moderator: What motivated you to apply the recommendations when feeding your young child?

Respondent 7: when I received these recommendations, I used them based on my knowledge then I realized there is a change compared to how my son was before is now strong and full of vigor.

Respondent 3: what motivated me was firstly, I looked into what they were asking me to do, I realized there was no harm in them. Secondly all what do asking me to do was not to do for them to benefit from what for my children to benefit from it. So that motivated me a lot concentrate on whatever thing they do or say.

Respondent 8: there is a boy that I usually see my neighborhood. The moment you see him, he is normal and is so chubby and strong so when one sees him, you like to carry him and call him chubby but among the foods we have seen that such children are fed with, we can also purchase them and use. Example is cassava. I least expected to see it make the list of foods. But surprisingly, it is there because I disregard it because it is only starch that is contained in it. I started practicing this and now my child is becoming chubby too. “---Fathers FGD

“MODERATOR: What gave you the courage to try these advices?

Respondent 7: Anytime they came and gave advice immediate I call the father and tell him, he will buy all they ask us feed the child with it. When you try is for a while and notice the child has changed then you keep giving him. “---Mothers FGD

**Feeding bowl:** N/A

**Pamphlets**: N/A

**Phone calls/text messages:** N/A

**Posters:** N/A

**Radio/TV/social media:** N/A

**Rec. Dietary pattern:** N/A

**Religious Leaders:** N/A

**What were the differences?**

**CBOs:** N/A.

**CHEWs:** Mothers did not report motivations from CHEWs. Only fathers did.

Moderator: What motivated you to apply the recommendations when feeding your young child?

Respondent 7: when I received these recommendations, I used them based on my knowledge then I realized there is a change compared to how my son was before is now strong and full of vigor.

Respondent 3: what motivated me was firstly, I looked into what they were asking me to do, I realized there was no harm in them. Secondly all what do asking me to do was not to do for them to benefit from what for my children to benefit from it. So that motivated me a lot concentrate on whatever thing they do or say.

Respondent 8: there is a boy that I usually see my neighborhood. The moment you see him, he is normal and is so chubby and strong so when one sees him, you like to carry him and call him chubby but among the foods we have seen that such children are fed with, we can also purchase them and use. Example is cassava. I least expected to see it make the list of foods. But surprisingly, it is there because I disregard it because it is only starch that is contained in it. I started practicing this and now my child

**Feeding bowl**: N/A

**Pamphlets**: N/A

**Phone calls/text messages:** N/A

**Posters:** N/A

**Radio/TV/social media:** N/A

**Rec. Dietary pattern:** N/A. Fathers did not report any motivations to the recommended dietary pattern. Only mothers did. Some mothers said that they were motivated to try the foods because they were curious to “see the importance of eating the foods”

“Moderator: Why did you try these advice?

Respondent 10: to see the importance of eating this food”---Mothers FGD

**Religious Leaders:** N/A

**Any other thoughts you would like to share?**

**Ward name: Turunku**

*Note: For each of the questions, note if it was similar or different between the groups of fathers and mothers from each ward. Include any quotes that are particularly relevant*

**PERCEPTIONS**

Ques 1: How did fathers/mothers in the area describe their thoughts/opinions/likes and dislikes (**perceptions)** of the recommendations made by the program (CBOs, CHEWs, Religious leaders, phone calls/text messages, pamphlets, posters, feeding bowl, rec. dietary pattern) about feeding their children?  **Hint: look out for use of Perceptions + Exposure codes**

**What were the similarities?**

**CBOs:** Mothers did not report perceptions with CBO’s. Only Fathers did.

**CHEWs**: Both Mothers and Fathers expressed that they liked the advice given by CHEWs. They mention that the advice they got was useful and felt that it was good for their children and their family. Some fathers spoke about how the advice from the CHEWs served as reminders to them about the benefits/advantages in feeding their children particular foods. One of the mothers spoke of the credibility of the CHEWs and mentioned how much she trusted them to give her advice which will not be harmful to her children.

“Moderator: What is your opinion on the advice?

Respondent 8: We know they are trained to advice us on child feeding and when we tried it the outcome was wonderful. What made us understand what they explained to us is because they do not just enlighten us about child feeding they also enlighten us about hygiene. The hygiene of ourselves, our children and that of our household. Because if you give your child all these things and you do not keep the clean or keep the food the child eats clean, it will not build the child’s body. We are really grateful for the explanations given to us.

Respondent 5: I plead to everyone to make use of the advice because our baby will look different for other. My opinion is please because of God we want to continue with these advises they gave us because it is very relevant to our children. And even you the mother, if you child gets such kind of care, the child will look different from other children that do not get such kind of care.”

“Moderator: Is there any advice given you like or dislike?

Respondent 3: All the advice is good. All the advises they have given us, we like them. We are grateful because they have come to enlighten us about things we do not know.”

“Moderator: What is your opinion on the health workers coming to your houses to advice you on child feeding?

Respondent 5: I wish they will continous coming and give other advices on child feeding. Our opinion is that they should continue coming to give us these advises. It makes us happy and we see its relevance.

Respondent 2: I like the advice they gave us is good and we are happy seen our child having good growth” ----- Mothers FGD

“Moderator: What is your opinion of those recommendations?

Respondent: Their recommendations are true and we have discovered that it is good and our children and families. Because they go to female parents and advise them on how to care for their family’s health, for the pregnant ones they tell them how to deliver safely and that when they put to bed, they should feed the child breastmilk because all the essential nutrients recommended for children are all contained in the breastmilk. It is a good thing as we really need it. It makes us happy and we are grateful.”

Moderator: What is opinion about having CHEW talking about child feeding during their visit to your home?

Respondents: It is very good and important thing they are doing. It serves as a reminder and the remainder is very important. It is something you know but are ignorant of its benefits. You the health person know it but the person you are telling needs it more. It is a good thing we hope for and are grateful about.” ----- Fathers FGD

**Feeding bowl:** N/A

**Pamphlet**: Both Mothers and Fathers expressed positive opinions on the pamphlets. They said that they were good guides that showed the proper way to feed their children.

“Moderator: What did you like about these flyers?

Respondent 10: I like the way she is breast feeding her baby? I like the way the mother is breastfeeding the child the right way.

Respondent 7: It serves as reminder to mothers. We like it because when we forget, we can carry it and remember what we were told.” ----- Mothers FGD

“Moderator: What did they recommend? What is your opinion of these recommendation?

Respondent 1: The kind of advice we do give about feeding children with nutritious food are: We observed that some of us have the kinds of nutritious foods they talked about but don’t know how to use them, we do show them and tell them that they do not need to go to the market to buy it. We got sensitized, we learnt and we were given posters and pamphlets and plates to help guide us.” ----- Fathers FGD

**Phone calls/text messages**: Fathers mention that the text messages are reminders to them about what to feed their children and how to use those appropriately. Both groups mention that they think the messages are important and that they should continue to be sent.

“Moderator: Where did you see/heard these advice and what did you like about it?

Respondent 9: Everything about the advice is what I like. Well truly I didn’t get these advises on radio or television. They came to our houses to sensitize us and we are very grateful about that because before when I give birth to a child they start giving the child hot water from the first day and even the second day. They will not even give the child breast milk, they will say no, give the child water first because that is what the child needs. But because of the enlightenment, I now give my child only breast milk without water at all. I do not breastfeed him and the milk will be pouring out of his mouth and air enters his mouth. I make sure I hold it well for him to feed well.

Respondent: what I want to say is that they should continue going house to house to sensitize us so that those that haven’t heard or tried it can start.

Respondent: there is nothing we do not like. All we have to say is we are grateful and may God reward you and increase your position.” ---- Mothers FGD

“Moderator: What is your opinions of the messages themselves?

Respondents: The messages are very important. They should continue sending them.

Moderator: What is your opinion about receiving messages on young child feeding through your phone?

Respondents: The messages are always welcomed and they should continue sending it.” ---- Fathers FGD

**Posters:** Both Mothers and Fathers expressed that they like the posters. Fathers mentioned that the visual effects of the pictures were attractive and made them stop to notice what was being communicated. Other fathers also mentioned that the size of the posters and the use of pictures(ie large) communicated to them the messages the posters contained were important and therefore made them stop to read.

“Moderator: What did you dislike about these posters?

Respondent 2: Nothing. We like everything” ----- Mothers FGD

“Moderator: What do you like about this?

Respondent: I like how it explains things clearly. Reminder. It draws our minds back because when you see a plate of food and picture, and because it takes about food, you will want to read it. Because of how big it is and it contains pictures, when you see it from afar it will attract your attention and you will want to read it. It shows the message is of great importance”

“Moderator: What do you like about this poster?

Respondents: we like everything about it. We like how they use pictures to present ideas and guide both fathers and mothers on breast feeding and child feeding.

Moderator: What do you not like about the poster?

Respondents: Nothing

Moderators: Do you think this poster is meant for people like you?

Respondents: It is for both us, the father and the women, the mothers.

Moderator: What do you think about this advice?

Respondent: These advises as explained to remind parents, with those providing and those preparing on how to progress. Stopping of all these things will set us back

Respondents: It makes understand that the Icare Foundation is really concerned about the health and well-being of young children. And it will help parents and it is something we like because they are concerned about small children’s Health.” ----- Fathers FGD

**Radio/TV/social media: Only mentioned in the mother FGD**

**Rec. Dietary pattern:** N/A

**Religious Leaders:** Both Mothers and Fathers expressed positive opinions on advice from religious leaders. Both express a lot of trust in religious leaders. Mothers spoke of how the advice from the religious leaders were encouraging and motivating to their husbands and fathers were more adherent especially because they were a trusted source. Fathers spoke indepth about the credibility of the religious leaders and that backing the feeding advice they give with scripture makes it even more credible, that they “do not lie” and that what they “say is final” because they “know better”.

“Moderator: What is your opinion on religions leaders discussing child feeding during meeting?

Respondent: It will give the courage to support them. It will further motivate our husbands. If we tell them and they do not do it, if they give hem sermons or they hear it on radio or religious leaders it will encourage them more than we telling them.

“Moderator: What did you like and dislike on religions readers discussing child feeding?

Respondent 7: They will explain better than the way the mother will explain to them. Honestly, we want them to talk so that they can know their responsibilities. They will listen to the elders more than us.” ---- Mothers FGD

Moderator: What is your opinion about having religious leaders talk about child feeding during their sermons?

Respondents: We are very happy to have them talk about it. Imams are leaders in religious aspect. They know better, so when they talk, what they say is final. They are the fathers of the community and everything they do concerns Faith. That is why when they talk, people quickly listen.” ---- Fathers FGD

**What were the differences?**

**CBOs:** Only Fathers reported perceptions on CBOs. Most fathers expressed positive thoughts about what they learnt from the CBOs. Only one of the fathers mentioned an initial negative reaction (being slightly offended) to being taught how to feed his children correctly but he also said that after realizing how much he was learning, these feelings were replaced with appreciation of how much was being taught him.

“Moderator: What did they recommend? What is your opinion of these recommendation?

Respondent 1: The kind of advice we do give about feeding children with nutritious food are: We observed that some of us have the kinds of nutritious foods they talked about but don’t know how to use them, we do show them and tell them that they do not need to go to the market to buy it. We got sensitized, we learnt and we were given posters and pamphlets and plates to help guide us.”

“Moderator: What is your opinion about having someone talk with fathers about child feeding?

Respondent 8: At first, I thought it was a great disrespect to me for someone to teach me how to feed my child but I later realized that there is so much I need to learn and so much I don’t know so I decided to put my mind and learn and I have also been working with what I have learnt.

Respondent: What I will say is, the recommendations were very important because it has been fully embraced in the community and neighboring communities. It is no longer a strange topic. People know a lot about it and it has been useful.” ----- Fathers FGD

**CHEWs**: One father mentioned that in certain instances the strategies adopted by the CHEWs to target just one wife of a polygamous family was breeding some hostilities in certain households. One of the mothers mentioned how some women would refuse to listen to the advice of the CHEWs on foods to feed their children because they felt that the advice was not accompanied by food/monetary support from the CHEWs. She went on further to say that CHEWs should encourage fathers to be more supportive in the provision of nutritious food for feeding children.

“Moderator: What is opinion about having CHEW talking about child feeding during their visit to your home?

Respondents: It is very good and important thing they are doing. It serves as a reminder and the remainder is very important. It is something you know but are ignorant of its benefits. You the health person know it but the person you are telling needs it more. It is a good thing we hope for and are grateful about.

Respondent: there was a time they came like I said earlier, they met a woman breastfeeding her baby. Like I told them, when breastfeeding makes to not let air enter the breast or the baby’s mouth but they took it for granted and made it seem like I do not know what I am doing. So when those women came, they took the child and took him close to the breast and told her that, if she lets air enter the child’s stomach, the child will fall sick and have some ailments but if you bring the child close, the child will be healthy. They now agree that the training they gave us is really important and the reminder is also very good.

Respondent: I met them while entering the house before giving out some plates to parent showing them how to use it. The lines that a 6-9 months old food should stop. They were showing them how to use it. They give our wives these plates but when they enter some household that have more than one wife that have children between 6-9 month and give just one plate, they face issues because they women get angry and say why will you give her and not give me too. I have met that truly before”--Father FGD

“Respondent 3: They should increase the way the pass these messages for other people to know. What I like is that when they come and some women refuse, till tomorrow some women will say that they will not provide it for them so why will they disturb them like this. So they should sensitize them and tell them to not wait for someone to provide it, it is for their children’s health. They should also advise the father to put in more effort when providing nutritious foods for the family

Respondent: what I want to say is that they should continue going house to house to sensitize us so that those that haven’t heard or tried it can start.”--Mothers FGD

**Feeding bowl:** Mothers express that they like the feeding bowl/plate and say it is helpful for determining how much children should be fed based on the calibrations in the bowl. The only thing Fathers mention is that they have seen occasions where in a household with more that one wife, the women tend to get offended if a CHEW gives one wife gets the feeding bowl and not the other.

“Moderator: What did you like about the plate?

Respondent 10: The indication inside the plate for each month that indicates the quantity of food to feed the child.

Moderator: What did you dislike about the plate?

Respondent 8: Nothing. I like everything about the plate because if you look at it, it is not just any plate it has things that you are supposed to be feeding your child. That is why I like the plate.

Respondent 9: Nothing. I like everything.” ---- Mothers FGD

“Respondent: I met them while entering the house before giving out some plates to parent showing them how to use it. The lines that a 6-9 months old food should stop. They were showing them how to use it. They give our wives these plates but when they enter some household that have more than one wife that have children between 6-9 month and give just one plate, they face issues because they women get angry and say why will you give her and not give me too. I have met that truly before.” ----- Fathers FGD

**Pamphlet**: N/A

**Phone calls/text messages**: Mothers mentioned how they wanted the messages to be sent to fathers more so that they will help out.

“Moderator: Where did you see/heard these advice and what did you like about it?

Respondent 3: They should increase the way the pass these messages for other people to know. What I like is that when they come and some women refuse, till tomorrow some women will say that they will not provide it for them so why will they disturb them like this. So they should sensitize them and tell them to not wait for someone to provide it, it is for their children’s health. They should also advise the father to put in more effort when providing nutritious foods for the family” ---- Mothers FGD

**Posters:** N/A

**Radio/TV/social media:** N/A

**Rec. Dietary pattern:** N/A

**Religious leaders:** Mothers and fathers differed on the reasons they liked advice from religious leaders. Mothers mentioned that they liked advice from Religious leaders because fathers would listen to the religious leaders instead of them. They say that they would like grandparents to receive advice from religious leaders as well so that they understand that the advice they are using is actually good for the baby. On the other hand, fathers talk more about the aspect of trust and how they believe that religious leaders will give them proper advice.

“Moderator: What did you like and dislike on religions readers discussing child feeding?

Respondent 7: They will explain better than the way the mother will explain to them. Honestly, we want them to talk so that they can know their responsibilities. They will listen to the elders more than us.

“Moderator: What will you say on the advice about your family?

Respondent 9: We wish religions leaders will enlighting our grand parent more because they never believe we should give our baby breast milk for 6month. We want religious leaders to educate the parents and grandparents of the children. Some do say we breastfeed children till 6month, they say the breast milk is hot and you are cheating the child by not giving them water. So maybe when they enlighten people on how the child will feel they can help stop people from giving them water while breasting and let them know you are not cheating them.” ----- Mothers FGD

“Moderator: What is your opinion of those recommendations?

Respondents: We agree with them and we trust what they say. The ways followed are really very important ways. You see religious leaders play a huge role and people know that they cannot tell lies. Imam will not lie in the Mosque and Pastor will not lie in the church. Isn’t that true? As long as it comes out from their mouths and they give reference with scriptures and pastor too will do same, it is something that parents take gradually, because people’s rate of assimilation is different. What somebody will understand immediately, someone else wouldn’t? It is something that we have tried and seen results.” ----- Fathers FGD

**EXPERIENCES**

Ques 2: What were fathers/mothers’ thoughts on the things which worked/didn’t work **(experiences**) and the reasons raised for trying/not trying the recommendations made by the program (CBOs, CHEWs, Religious leaders, phone calls/text messages, pamphlets, posters, feeding bowl, rec. dietary pattern)? **Hint: look out for use of Experiences + Exposure codes**

**What were the similarities?**

**CBOs:** Fathers did not report experiences with CBOs. Only mother did.

“Moderator: What differences did you experiences?

Respondent 6: He bought lot of food for the baby and at times he feeds the baby himself

Respondent 9: Before whatever he eats is what is fed to the child but now once the baby clock 6 months. He will buy what is require by the health workers and even feed the baby himself” ---- Mothers FGD

**CHEWs:** Both Mothers and Fathers reported positive experiences with recommendations from CHEWs related to less frequent illnesses in their children, children growing healthy and strong and being less prone to fall.

“Moderator: What will you say after trying these advices on child’s feeding

Respondent 6: We will say thank you very much. Glory be to God. This advice they have given, honestly, I have tried it on my daughter and glory be to God I have seen improvement because my daughter is now healthy, strong, wise and have stopped falling since unlike before when I wasn’t giving her all these things. Honestly, I have tried it and seen improvement. Glory be to God.

Respondent 4: We will say thank you very much. If you breast you child with just breast milk for 6 months, you child will not fall sick frequently and even the child’s skin will change” ----- Mothers FGD

“Moderator: What are your experiences with trying to apply the recommendations in your families?

Respondents: Applying their recommendations is worth it and it has helped a lot of mothers, fathers and their children.” ----- Fathers FGD

**Feeding bowl:** N/A

**Pamphlets**: N/A

**Phone calls/text messages:** N/A

**Posters:** N/A

**Radio/TV/social media:** Only mothers reported experiences with media. One of the mothers mentioned seeing differences in the growth of her two children: the preceding child was frequently ill but the current child with whom she followed the feeding recommendations from the radio/tv is less frequently ill

“Moderator: Is there changes in the way you feed your baby after listing to these advice?

Respondent 5: My first child always feels sick but now my second baby is healthy and strong with these advices. Surely there have been changes because of what they came to tell us. The child I nursed at first fell sick so much but when I had my second child and they came to sensitize us and I practiced it, my child is healthy and strong.

Respondent 2: All the advice is for our sake because they are all good. Since they came to tell us and we practice it, we have been very happy and it is expected of a mother to give her child what she is supposed to be giving him”---Mothers FGD

**Rec. Dietary pattern:** Only mothers reported experiences with the recommended dietary pattern and they were positive.

“Moderator: Did you try this advice?

Respondent 2: Yes, we tried it very well.” ----Mothers FGD

**Religious Leaders:** Mothers did not report experiences with religious leaders. Only Fathers did. They mentioned positive results after application of the advice from the religious leaders. They also mentioned how much they take their responsibilities as providers more seriously.

“Moderator: what are your experiences with trying to apply the recommendations in your families?

Respondents: We have seen good results with our own eyes after applying the recommendations. What I can say about the recommendation given is that all the recommendations are good. We know somethings before but because of this program we have learnt a lot. This program has made us take it with high importance because you that your family’s responsibility is on you then you now have someone to help you. If you are sensible, you are supposed to take it seriously and be grateful to the person. Even in the religion, it is good to remind people. Human beings need to be reminded. Imams reminds us on Fridays and the Pastors remind people on Sundays. It is something that is in the book. It is something that God told the Prophets. But they try to remind the public and if you have forgotten, you will remember. It makes us happy and we are grateful about it.” ----- Fathers FGD

**What were the differences?**

**CBOs:** Only Mothers reported experiences on CBO’s (quotations listed under similarities)

**CHEWs:** N/A

**Feeding bowl**: N/A

**Pamphlets**: N/A

**Phone calls/text messages:** N/A

**Posters:** N/A

**Radio/TV/social media:** N/A

**Rec. Dietary pattern:** N/A

**Religious Leaders:** Only Fathers reported experiences with religious leaders (quotations listed under similarities)

**MOTIVATIONS**

Ques 3: How did fathers/mothers describe what motivated them to apply the recommendations from the program (CBOs, CHEWs, Religious leaders, phone calls/text messages, pamphlets, posters, feeding bowl, rec. dietary pattern)? **Hint: look out for use of Motivations + Exposure codes**

**What were the similarities?**

**CBOs:** Mothers did not report any motivations regarding CBOs. Only Fathers did. The fathers mentioned that some of the motivations to try out the advice from the CBOs were related to the fact that foods recommended are available locally and have health benefits which are being mentioned as well.

“Moderator: What motivated you to apply the recommendations when feeding your child?

Respondent 9: I was motivated to apply the recommendations when I realized how important they are. What motivated me to try this is that, the things are easy to get and it concerns health. We hear it on radio and other medium of transmission of information. We have this foods but we use it waste them because we didn’t its importance. If you come to our community to look for Moringa, they will fill you boot to the brim with it. That was how we used to do. Now that we know its importance, the moringa is now far from us. So, these things have motivated us. I have heard a Doctor talk about pumpkin. The importance of what only pumpkin can do to your body. And now I am now hearing about it again, then I agree that it is really very important.”

**CHEWs:** Fathers did not report any motivations regarding CHEWs. Only Mothers did. Mothers spoke about how their previous experiences with raising children has included losing some of the children due to sickness. They were therefore motivated to try the advice from the CHEWs by those negative experiences.

“Moderator: What give you courage to try these advices?

Respondent 7: Baby die at young age now and nobody will want to loss the baby that is why I stick to their advices. What encouraged us is because children always fall sick and lives are lost a lot. You wouldn’t want to give birth to a child and the child should die and leave you. That is why we have tried it and it has worked.

Respondent 4: The advice work for me very well. We have tried it and see that it is useful.

Respondent 8: I tried the advice on my girl and she is looking strong and healthy. My opinion is that we have tried it and seen its benefits and have seen its importance because I tried it with my daughter and I have seen how its usefulness.” ----- Mothers FGD

**Feeding bowl:** N/A

**Pamphlets**: N/A

**Phone calls/text messages:** N/A

**Posters:** N/A

**Radio/TV/social media:** N/A

**Rec. Dietary pattern:** N/A

**Religious Leaders:** Both Mothers and Fathers express that they are motivated to follow advice of religious leaders as they see improvement in their children’s health.

“Moderator: What give you the courage to try these advices on child feeding?

Respondent 6: Because of the health of the child. I feel encouraged because of my child’s health and the child no longer frequently vomit, purge or fall sick anymore. If you adhere to the advice they give you, you will notice that the child will be very healthy.” ----- Mothers FGD

“Moderator: What motivated you to apply the recommendations when feeding your child?

Respondents: We have realized that it is good for us, our children and our families. What motivated us is that, whatever they remind you of and advised about, if you practice it, it will be such a blessing, it will improve the health of your family and you too. It is compulsory for you to try the advice and it motivates on because whatever concerns health is highly important and should be treated as one.” ----- Fathers FGD

**What were the differences?**

**CBOs:** Only Fathers reported motivations regarding CBOs (quotations listed under similarities)

**CHEWs:** Only Mothers reported motivations regarding CHEWs (quotations listed under similarities)

**Feeding bowl**: N/A

**Pamphlets**: N/A

**Phone calls/text messages:** N/A

**Posters:** N/A

**Radio/TV/social media:** N/A

**Rec. Dietary pattern:** N/A

**Religious Leaders:** N/A

**Any other thoughts you would like to share?**

**Ward name: Zango Aya**

*Note: For each of the questions, note if it was similar or different between the groups of fathers and mothers from each ward. Include any quotes that are particularly relevant*

**PERCEPTIONS**

Ques 1: How did fathers/mothers in the area describe their thoughts/opinions/likes and dislikes (**perceptions)** of the recommendations made by the program (CBOs, CHEWs, Religious leaders, phone calls/text messages, pamphlets, posters, feeding bowl, rec. dietary pattern) about feeding their children?  **Hint: look out for use of Perceptions + Exposure codes**

**What were the similarities?**

**CBOs: N/A**

**CHEWs**: both mothers and fathers mentioned positive thoughts about the advice received from the CHEWs. Some fathers said they would like even more visits so that other members of their families could learn more from the CHEWs about child feeding.

“Moderator: If not, would you like the CHEW to schedule visits when you are home?

Respondent 8: Yes, by the grace of God. When they came and met with our wives at home, they gave them vital information about how to feed their young children. They even gave those plates and how to feed children. We are suggesting that they come back again to those houses and do more enlightenment on child feeding practices that will be valuable to the children, Hygiene.

Respondent 4: We will like them to come back and do more teaching especially to those houses that they didn’t visit the last time they came. Personally, I would like if they visit my house so that I will learn more and my wife and other members of the family too will be enlightened on the necessary information they carry.”---Fathers FGD

**Feeding bowl: N/A.** Only mothers had perceptions about this. See below

**Pamphlet**: Both mothers and fathers had positive thoughts about the pamphlets. Some fathers specifically mentioned liking the illustrations used in the pamphlets.

“Moderator: What do you like about the pamphlet?

Respondent 2: What I like here are the diagrams and the explanations of each one of them. How to fed breast milk, how the mother feeds the child with variety of food for children 6-23 months.

Moderator: What do you not like about this pamphlet?

All Respondents: None.

Moderator: Do you think this pamphlet is made for people like you? Why or why not?

All Respondents: Yes, it is made for us.

For participants seeing the pamphlet for the first time.

Moderator: What is this pamphlet asking you to do?

Respondent 9: It shows us that our wives that are pregnant should feed on some particular foods that are very nutritious till she puts to bed which has been a great to lesson to us.

Moderator: What do you think about this advice?

Respondent 4: It is very good because it is very educative.”--- Fathers FGD

**Phone calls/text messages**: Only fathers had perceptions about this. See below

**Posters:** Both mothers and fathers mentioned positive things about the posters. However there were some slightly different things mentioned as written below

**Radio/TV/social media:** None mentioned in the focus groups for this LGA

**Rec. Dietary pattern:** N/A. Mentioned only by mothers

**Religious Leaders:** N/A. Mentioned only by mothers

**What were the differences?**

**CBOs:** Only fathers mentioned perceptions regarding CBOs. Fathers said that they constantly encouraged other fathers to take seriously the IYCF messages shared at the CBO meetings because they had found them to be beneficial. Also, they had encouraged other people to participate in similar groups in the community in order that they are able to access similar information which is beneficial to them.

**“**Moderator: What is your opinion about having someone talk with fathers about child feeding during CBO meetings? (Probe) on what they liked and didn’t like.

Respondent 7: When we come, we usually tell people to give much importance and priority to whatever information they receive because in the long run, it will benefit them and their children. For those who do not take these awareness campaign seriously, it would affect them in the long run. People try to join various group as such so that they are always be in the light of the happenings around them. In fact, like my group, those who are in attendance this week will still attend the following week including some new ones who joined up. That was how we influenced their decision of joining these groups.” Fathers FGD

**CHEWs**: N/A

**Feeding bowl:** Only mothers had perceptions about the feeding bowl and they were all positive. Some mothers spoke about the bowl being helpful in reminding them what to feed their children with since they see those foods being displayed on the feeding bowl

“Moderator: What do you like about this bowl?

Respondent 8: This bowl is helpful; we make sure when we want to feed our child, we wash their hands before eating

Respondent 7: The bowl has helped us, we feed them from the bowl and after eating, and we wash the bowl and keep it in a clean place. And what we see on the bowl are what we try to feed them with when God provides us what to give them.

Respondent 10: The children are happy whenever we serve them food with the bowl, they like it so much

Moderator: What do you not like about this bowl? Is there anything we don’t like about this bowl?

All Respondents: Nothing

Moderator: Thank you”—Mothers FGD

**Pamphlet**: N/A. There were no differences between fathers and mothers about their general perceptions of the pamphlets. See above

**Phone calls/text messages**: Fathers had positive thoughts about the phone calls/text messages. They mentioned the convenience of being able to share the messages easily because they were in text message form and so they could be forwarded to other people.

“Moderator: What is your opinion of the messages themselves? (probe/prompt with part of message) on what they liked and didn’t like.

Respondent 1: To me, these messages are still a way of passing the information across to people if in case one didn’t hear it either from the groups or in the mosques from the imam, or the information did not reach one’s community or his house was not visited. But with the text anywhere you are you will receive it at your own comfort and convenience.

Respondent 5: It is a good thing because it comes to me and educated me on the things I did not know before, these messages I receive now have educated me and I know a lot of ways and how to provide for the family to ensure that they are well fed with nutritious foods which will enhance their growth.

Respondent 7: I have seen the importance of these messages because it has changed my perception about how children are fed. My little child will mention some things that she wants to take then I will tell her “Okay, but my pocket is empty” (No money), you will be have to be patient. I really like receiving text messages, I really do.”---Fathers FGD

**Posters:** although both mothers and fathers mentioned positive thoughts about the posters, there were slight differences in what were mentioned. Some mothers said they liked that the images in the posters depicted fathers feeding their children. Some fathers on the other hand said that they liked that posters presented varieties of foods. Other fathers also said that they would have desired to see what particular foods are recommended to feed their children depicted in the posters as well as the timing of feeding those foods

“Moderator: What do you like about this poster?

Respondent: What we like here and what we would want to see here are those foods that we need to feed our children should be presented in pictures, so that we know some of them and when to feed such foods.

Respondent 10: What we like about the poster are the different foods and writings.

Respondent 1: The mother of the child is feeding the child with the foods, necessary for the child.

Moderator: What do you not like about the poster?

All Respondents: There is nothing (collectively answered)

Moderator: Do you think this poster is meant for people like you? Why or why not?

All Respondents: Surely (Collectively answered)”---Fathers FGD

“Moderator: What do you like about the different types of food in the poster?

Respondent 8: I saw the way the father is helping the mother to feed the child food

Respondent: we see that the father is helping the mother to feed the son or daughter.

Moderator: What do you not like about this poster?

All Respondents: Nothing

Moderator: How, if at all, has this influenced how you feed your 6 – 23 months old?

Respondent 5: Because of the way the child is looking healthy in the picture.”—Mothers FGD

**Radio/TV/social media:** None mentioned in the focus groups for this LGA

**Rec. Dietary pattern:** Mothers expressed general positive thoughts about the recommended diets

“Moderator: What do you think about this advice?

Respondent 6: It is a good recommendation.

Respondent 4: It will make them healthy and strong because the combination contains vitamin C in it

Moderator: Anyone else? Thank you”--- Mothers FGD

**Religious leaders:** Mothers expressed positive thoughts about the advice fathers received from religious leaders.

“Moderator: What do you like and didn’t like?

Respondent 5: I am happy with the information they gave to our husband that they should care for their family and I am happy with that.

Moderator: Thank you

Moderator: What is your opinion about having religious leaders talk about child feeding during their sermons?

Respondent 8: Since they informed our husbands and encourage them to care for their wives and children

Respondent 3: We appreciate them with the advice given to our husbands, God bless them

Moderator: Is there anything you like and didn’t like about what they are saying?

Respondent 9: I like all what they said because there is nothing I don’t like.

Moderator: What new information about child feeding did you learn from the sermon?

Respondent 10: We learnt a lot because we never practiced these recommendations with the other children but now we do apply the recommendations they were given to us and our husbands, we are grateful

Moderator: Is there anything you like and didn’t like?

Respondents: No”----Mothers FGD

**EXPERIENCES**

Ques 2: What were fathers/mothers’ thoughts on the things which worked/didn’t work **(experiences**) and the reasons raised for trying/not trying the recommendations made by the program (CBOs, CHEWs, Religious leaders, phone calls/text messages, pamphlets, posters, feeding bowl, rec. dietary pattern)? **Hint: look out for use of Experiences + Exposure codes**

**What were the similarities?**

**CBOs: N/A.** No experiences mentioned by fathers and mothers from this LGA

**CHEWs:** Both mothers and fathers expressed positive experiences with practicing the advice from the CHEWs. Some fathers spoke about seeing differences in the hygiene practices of mothers with respect to child feeding.

“Moderator: What are your experiences with trying to apply recommendations in your families? (Probe) on what works and did and add.

Respondent 1: The women accepted the recommendations especially on the feeding aspect where the mothers wash the hands of the children before they are fed and even the mothers themselves ensure to wash their hands before they feed their children. This was very acceptable because we have seen lots of changes in our homes. They are being careful of the hygiene in feeding, the house, the community and the utensils they use in preparing the food. Then, after meals the used dishes were kept until tomorrow but now, dishes are washed immediately after meals and take it into the kitchen and close it. Honestly, we have seen a lot of progress.”—Father FGD

**Feeding bowl:** N/A. No experiences mentioned by both fathers and mothers from this LGA

**Pamphlets**: N/A. No experiences mentioned by fathers and mothers from this LGA

**Phone calls/text messages**: Only fathers mentioned this. see below

**Posters:** N/A. No experiences mentioned by fathers and mothers from this LGA

**Radio/TV/social media:** N/A. No experiences mentioned by fathers and mothers from this LGA

**Rec. Dietary pattern:** Only mothers mentioned this

**Religious Leaders:** Some differences. See below

**What were the differences?**

**CBOs:** N/A. No experiences mentioned by fathers and mothers from this LGA

**CHEWs:** N/A. both mothers and fathers had positive experiences. See above

**Feeding bowl**: N/A. No experiences mentioned by fathers and mothers from this LGA

**Pamphlets**: N/A. No experiences mentioned by fathers and mothers from this LGA

**Phone calls/text messages:** fathers mentioned being able to be flexible in their provision of resources for child feeding as a positive experience for them

“Moderator: What are your experiences with trying to apply the recommendations from the messages in your families? (Probe) on what worked and what didn’t work and why they did or didn’t try the recommendations.

Respondent 1: We try our best as Respondent 7 said, “It is not every time one must do or provide”. At least the father can do well to provide at least twice or thrice in a week. Also, if the father is buoyant enough, he can provide for a longer duration of time so that it will be readily available for probably a month or more.” ---Fathers FGD

**Posters:** N/A. No experiences mentioned by fathers and mothers from this LGA

**Radio/TV/social media:** N/A. No experiences mentioned by fathers and mothers from this LGA

**Rec. Dietary pattern:** A large majority of mothers mentioned not having yet applied the recommended food combinations.

“Moderator: Have you tried this advice?

Respondent 9: We have tried it, we work with their recommendations.

Moderator: Who else have tried this recommendation?

And those of you that have not tried this recommendation, why have you not tried it?

Respondent 9 said she tried it, why have you not tried it

Moderator: Respondent 10, why have you not tried it?

Respondent 10: God has not given me the opportunity

Respondent 8: God has not given me the opportunity to try it that time.

Respondent 7: Because of the situation on ground and also because of the COVID 19. That is why

Respondent 6: Because of the situation and hardship in town

Respondent 5: God has not given me the opportunity to try it

Respondent 4: Because of the situation in town but we are asking God to help us, so that we can try it

Respondent 3: God has not given me the opportunity

Respondent 2: Because of the situation in town

Respondent 1: God has not given me the opportunity to try it but only tried the ones that were easy for me”---Mothers FGD

**Religious Leaders:** Mothers mentioned general positive experiences with applying the recommendations and said that all the advice was good. Fathers on the other hand spoke about logistics related cautions which the Imams gave them when they were working with the Imam’s on the campaign

“Moderator: What are your experiences with trying to apply the recommendations in your families? (Probe) on what worked well and didn’t work well and why they did and didn’t try the recommendation.

Respondent 2: When the Imam told us to fear God as members to this group willing to sacrifice and work, then we should do it with all honesty and sincerity. If we can’t do all that the work entails, then it is better we leave the work than to take it up and become unfaithful to the whole work. If someone knows the work does request to be paid before he will do it is not willing to sacrifice and when he collects the money and uses it for his gain, he will surely pay someday in the long run.”---Fathers FGD

“Moderator: What are your experiences with trying to apply the recommendations in your family?

Respondent 5: All we have to say is thank you, may God bless you.

Moderator: Anyone else?

Respondents: No

Moderator: What worked and didn’t work and why you did or didn’t try the recommendations?

Respondent 9: All I have to say is God bless then and increase them in knowledge.

Moderator: Is there anything that didn’t work?

Respondent 5: There is nothing, all the advices were good and we thank you and may God bless their kindness with blessings.

Moderator: Thank you”---Mothers FGD

**MOTIVATIONS**

Ques 3: How did fathers/mothers describe what motivated them to apply the recommendations from the program (CBOs, CHEWs, Religious leaders, phone calls/text messages, pamphlets, posters, feeding bowl, rec. dietary pattern)? **Hint: look out for use of Motivations + Exposure codes**

**What were the similarities?**

**CBOs: N/A.** Only fathers spoke about this

**CHEWs:** N/A. Only mothers spoke about this

**Feeding bowl:** N/A. No motivations mentioned by fathers and mothers from this LGA

**Pamphlets**: N/A. No motivations mentioned by fathers and mothers from this LGA

**Phone calls/text messages:** N/A. No motivations mentioned by fathers and mothers from this LGA

**Posters:** N/A. No motivations mentioned by fathers and mothers from this LGA

**Radio/TV/social media:** N/A. No motivations mentioned by fathers and mothers from this LGA

**Rec. Dietary pattern:** N/A. No motivations mentioned by fathers and mothers from this LGA

**Religious Leaders:** N/A. Only mothers spoke about this

**What were the differences?**

**CBOs:** only fathers mentioned motivations with CBOs**.** Some of the fathers mentioned being motivated by the leadership roles they played in the campaign at the community level to apply the IYCF recommendations themselves in their homes. They said that it was important to be able to speak from experience of the benefits of the recommendations when sharing the messages with other fathers because it helped with the credibility of the messages as well as made others more encouraged/likely to apply them also.

“Moderator: What motivated you to apply the recommendations when feeding your young child?

Respondent 1: What motivated me in this campaign as a member of a group is that I personally and directly get involved in sensitising people in my community about the whole idea of this programme and not as it was before that we either hear from others or see other people doing it, but we are the ones directly involved in the work and so we made sure that our families are well informed and be the pace-setters to other families whom the good news also reached them. What you tell people to do should show on you first, then people will attest to it that it is not something to play with.”---Fathers FGD

**CHEWs:** Only mothers mentioned motivations in practicing the advice from the CHEWs. Some mothers spoke about how the children on whom they had applied the recommendations on were different in comparison to other children. They said that this motivated them to continue applying the recommendations

“Moderator: What motivated you to apply the recommendations when feeding your child?

Respondent 8: The children we applied these recommendations on them, there is a very big difference among them

Respondent 7: Applying these recommendations makes me happy the children are healthy and strong

Respondent 10: Our children are different from the other children whose parents did not apply this recommendation”---Mothers FGD

**Feeding bowl**: N/A. No motivations mentioned by fathers and mothers from this LGA

**Pamphlets**: N/A. No motivations mentioned by fathers and mothers from this LGA

**Phone calls/text messages:** N/A. No motivations mentioned by fathers and mothers from this LGA

**Posters:** N/A. No motivations mentioned by fathers and mothers from this LGA

**Radio/TV/social media:** N/A. No motivations mentioned by fathers and mothers from this LGA

**Rec. Dietary pattern:** N/A. No motivations mentioned by fathers and mothers from this LGA

**Religious Leaders:** Mothers spoke about the spiritual roles of the religious leaders in their lives as motivation for applying the advice received from them.

**“**“Moderator: What motivated you to apply the recommendations when feeding your child?

Respondent 7: We always pray to God for provision

Respondent 1: We are very happy, for this advice, coming from our, religious leader and they pray for us for provision and blessings (prayers) we are happy with these recommendations we pray to God to bless us all. And in this gathering may God bless us all and save us from the wickedness of humans.”----Mothers FGD

**Any other thoughts you would like to share?**
